# A draft nuclear-genome assembly of the acoel flatworm *Praesagittifera naikaiensis*

**DOI:** 10.1093/gigascience/giz023

**Published:** 2019-04-06

**Authors:** Asuka Arimoto, Tomoe Hikosaka-Katayama, Akira Hikosaka, Kuni Tagawa, Toyoshige Inoue, Tatsuya Ueki, Masa-aki Yoshida, Miyuki Kanda, Eiichi Shoguchi, Kanako Hisata, Noriyuki Satoh

**Affiliations:** 1 Marine Genomics Unit, Okinawa Institute of Science and Technology Graduate University, 1919-1 Tancha, Onna, Okinawa 904-0495, Japan; 2 Natural Science Center for Basic Research and Development, Gene Science Division, Hiroshima University, 1-4-2 Kagamiyama, Higashi-Hiroshima, Hiroshima 739-8527, Japan; 3 Division of Human Sciences, Graduate School of Integrated Arts and Sciences, Hiroshima University, 1-7-1 Kagamiyama, Higashi-Hiroshima, Hiroshima 739-8521, Japan; 4 Marine Biological Laboratory, Graduate School of Science, Hiroshima University, 2445 Mukaishima, Onomichi, Hiroshima 722-0073, Japan; 5 Department of Biological Science, Graduate School of Science, Hiroshima University, 1-3-1 Kagamiyama, Higashi-Hiroshima, Hiroshima 739-8526, Japan; 6 Marine Biological Science Section, Education and Research Center for Biological Resources, Faculty of Life and Environmental Science, Shimane University, 194 Kamo, Okinoshima-cho, Oki, Shimane 685-0024, Japan; 7 DNA Sequence Section, Okinawa Institute of Science and Technology Graduate University, 1919-1 Tancha, Onna, Okinawa 904-0495, Japan

**Keywords:** acoel, *Praesagittifera naikaiensis*, draft nuclear genome

## Abstract

**Background:**

Acoels are primitive bilaterians with very simple soft bodies, in which many organs, including the gut, are not developed. They provide platforms for studying molecular and developmental mechanisms involved in the formation of the basic bilaterian body plan, whole-body regeneration, and symbiosis with photosynthetic microalgae. Because genomic information is essential for future research on acoel biology, we sequenced and assembled the nuclear genome of an acoel, *Praesagittifera naikaiensis*.

**Findings:**

To avoid sequence contamination derived from symbiotic microalgae, DNA was extracted from embryos that were free of algae. More than 290x sequencing coverage was achieved using a combination of Illumina (paired-end and mate-pair libraries) and PacBio sequencing. RNA sequencing and Iso-Seq data from embryos, larvae, and adults were also obtained. First, a preliminary ∼17–kilobase pair (kb) mitochondrial genome was assembled, which was deleted from the nuclear sequence assembly. As a result, a draft nuclear genome assembly was ∼656 Mb in length, with a scaffold N50 of 117 kb and a contig N50 of 57 kb. Although ∼70% of the assembled sequences were likely composed of repetitive sequences that include DNA transposons and retrotransposons, the draft genome was estimated to contain 22,143 protein-coding genes, ∼99% of which were substantiated by corresponding transcripts. We could not find horizontally transferred microalgal genes in the acoel genome. Benchmarking Universal Single-Copy Orthologs analyses indicated that 77% of the conserved single-copy genes were complete. Pfam domain analyses provided a basic set of gene families for transcription factors and signaling molecules.

**Conclusions:**

Our present sequencing and assembly of the *P. naikaiensis* nuclear genome are comparable to those of other metazoan genomes, providing basic information for future studies of genic and genomic attributes of this animal group. Such studies may shed light on the origins and evolution of simple bilaterians.

## Background

Acoels are small, very simple, planaria-like animals lacking a coelom, a gut, and a circulatory system. Traditional taxonomy positioned the Acoela as the most basal order of the phylum Platyhelminthes [1]. Recent analyses using molecular data, however, have suggested that acoels are members of a new phylum, the Xenacoelomorpha, together with nemertodermatids and xenoturbellids [2–4]. However, whether Xenacoelomorpha is a monophyletic taxon, whether xenacoelomorphs are basal to all other bilaterians, and whether they have close affinity to ambulacrarians are matters of debate [2–4]. Nonetheless, acoels are pivotal to understanding the origins and evolution of bilaterians. Acoels also provide a platform for molecular studies of whole-body regeneration [5] and symbiosis with photosynthetic microalgae. Although mitochondrial genomes of 4 acoel species have been reported [6–8], their nuclear genomes have not been explored yet. Because nuclear genome information is essential to investigate biological questions regarding acoels, we sequenced and assembled a draft nuclear genome of the acoel *Praesagittifera naikaiensis* (urn:lsid:marinespecies.org:taxname:379972).

## Sampling and Sequencing

### Biological materials

**Figure 1:**
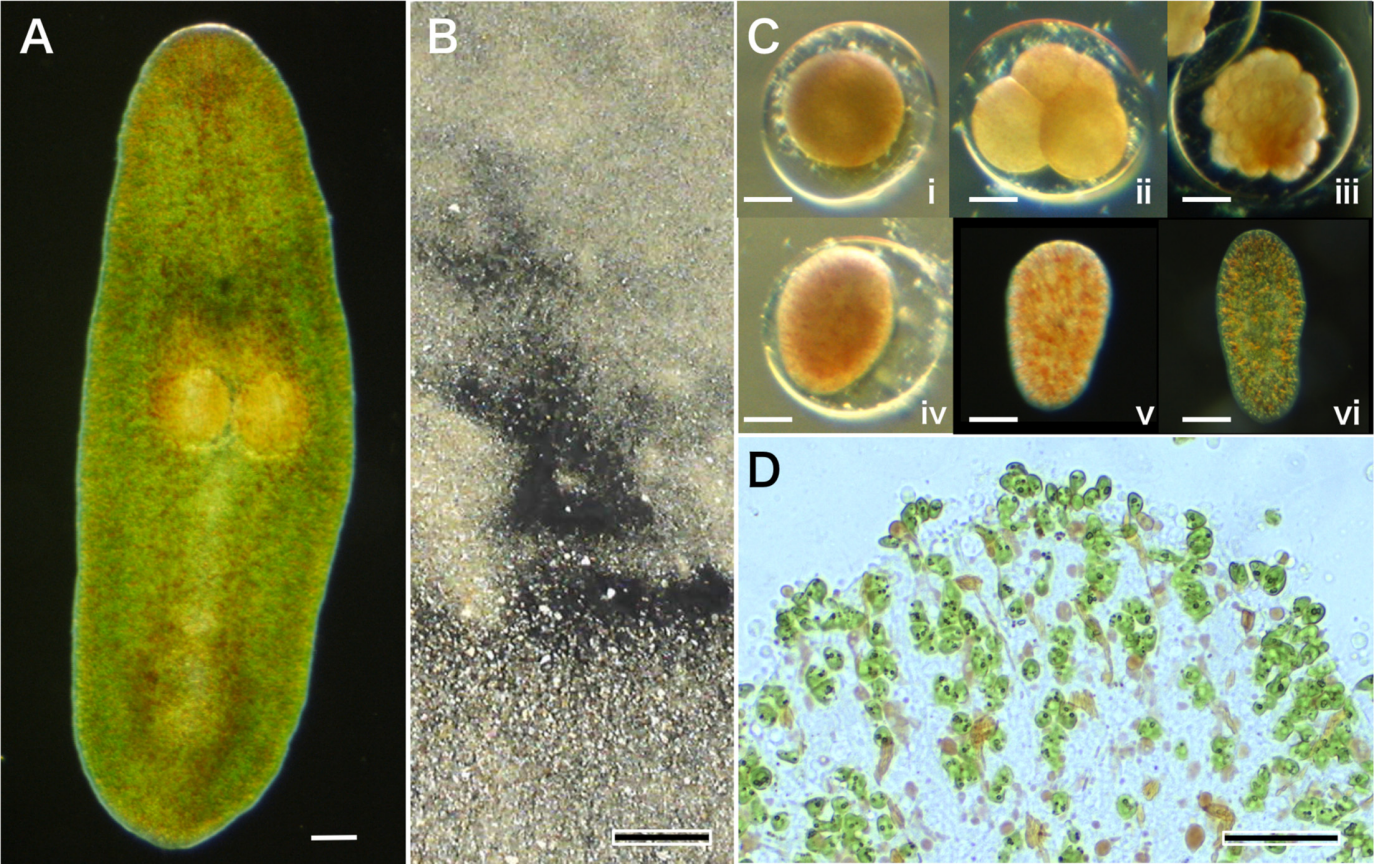
The acoel worm *P. naikaiensis*. (A) An adult, dorsal view. Anterior, top and posterior, bottom. Green dots throughout the entire body are symbiotic green algae. Two eggs are seen in the center of the worm. (B) An enormous number of adults gathering on the sandy seashore, resembling dark masses. (C) Embryogenesis. A newly laid egg within the eggshell (i), a 4-cell stage embryo (ii), a gastrula (iii), a flattened-stage embryo (iv), newly hatched aposymbiotic algae (v), and a symbiotic juvenile with symbiotic algae (vi). (D) A peripheral region of an adult worm showing symbiotic microalgae, *Tetraselmis* species. Scale = 50 μm in (A), (C), (D); 10 cm in (B).

The marine acoel worm *P. naikaiensis* is 2–3 mm in length (Fig. 1A) [9]. Members of this species are easily found at seashores of the Seto Inland Sea, Japan, especially during the early summer season (Fig. 1B). Adults contain symbiotic microalgae, *Tetraselmis* species, which are integrated during juvenile growth (Fig. 1C). Adults were collected at the seashore near the Marine Biological Laboratory of Hiroshima University and maintained in aquaria in the laboratory on a 12-h light/12-h dark photoperiod. Naturally laid eggs were collected and cultured for embryogenesis (Fig. 1C). Embryos were free of symbiotic microalgae. After washing embryos with filtered seawater, genomic DNA was extracted from them using the phenol/chloroform extraction method.

Embryos, juveniles, and adults were sampled for RNA sequencing. Total RNA extraction was performed using TRIzol Reagent (ThermoFisher, MA, USA, 15596-026) and an RNeasy mini Kit (Qiagen, Hilden, Germany, 74104).

### Library preparation and sequencing

#### DNA

All sequencing l**i**braries were constructed according to the manufacturers’ standard protocols. Briefly, for the Illumina platform, polymerase chain reaction (PCR)-free, paired-end libraries were prepared using an Illumina TruSeq DNA PCR-Free LT Library Prep Kit (Illumina, CA, USA, FC-121-3001). Four mate-pair libraries were prepared using a Nextera Mate Pair Library Prep Kit (Illumina, FC-132-1001) (Supplementary Table 1).

For the PacBio platform, a DNA library was prepared using the manufacturer's 10-kb (kilobase pair) template preparation protocol. A SMRTbell Template Prep Kit 1.0 (Pacific Biosciences, CA, USA, 100-259-100) was used for PacBio library preparation. The long-read DNA library was sequenced using a PacBio RSII sequencer employing P6-C4 chemistry (Pacific Biosciences, 100-372-700) with 360-min movie lengths. A total of 52 SMRT Cells were sequenced for long-read DNA library.

#### RNA

An RNA sequencing (RNA-seq) library was prepared using a TruSeq Stranded mRNA LT Sample Prep Kit (Illumina, RS-122-2101). The library was sequenced using the Illumina HiSeq 2500 platform (Supplementary Table 1).

Complementary DNAs for Iso-Seq libraries were prepared using a SMARTer PCR cDNA Synthesis Kit (Clontech, CA, USA, 634925). The SageELF size selection system (Sage Science, MA, USA) was used following the manufacturer's standard protocol (Supplementary Table 1). A SMRTbell Template Prep Kit 1.0 (Pacific Biosciences, 100-259-100) was used for Iso-Seq library preparation. The library was sequenced using a PacBio RSII sequencer employing P6-C4 chemistry (Pacific Biosciences, 100-372-700) with 360-min movie lengths. A total of 8 SMRT Cells were sequenced for the Iso-Seq RNA library.

### Assembly of mitochondrial and nuclear genomes

Adapter sequences in PCR-free and mate-pair Illumina reads were removed with Trimmomatic 0.36 (Trimmomatic, RRID:SCR_011848) [10] and NextClip 1.3.1 (NextClip, RRID:SCR_005465) [11], respectively. Low-quality (<Q20) inserts were removed using Sickle 1.33 (Sickle, RRID:SCR_006800) [12] after adapter cleanup. Reads that lacked a corresponding pair were discarded.

#### Mitochondrial genome assembly

To distinguish mitochondrial genome sequences from the nuclear genome assembly, we first assembled the mitochondrial genome. To this end, a mitochondrial 16S ribosomal RNA of *Symsagittifera roscoffensis* (accession No. NC_014578) [7] was used to collect PacBio long reads of *P. naikaiensis* sequences using BLAST+ 2.3.0 [13] with the “dc-megablast” option.

Collected reads longer than 1 kb and shorter than 12 kb were assembled using sprai 0.9.9.19 [14] with default settings. Circularity of assembled contigs was checked automatically in the sprai assembly pipeline.

#### Nuclear genome assembly

*P. naikaiensis* mitochondrial sequences were mapped onto trimmed reads using BWA 0.7.12 (BWA, RRID:SCR_010910) [15], and those read pairs that mapped onto the mitochondrial sequences were excluded from the data set. PacBio long reads were also mapped against the mitochondrial genome using BLASR (BLASR, RRID:SCR_000764) (commit version: 5.3.574e1c2) [16]. Only unmapped or cleaned-up Illumina and PacBio reads were assembled using the MaSuRCA (MaSuRCA, RRID:SCR_010691) assembler 3.2.2 [17].

Putative heterozygous and/or polymorphic sequences that remained in the assembled genome were merged as homozygous sequences using redundans 0.13c [18]. Gaps in the homozygous genome were filled using PBJelly (PBJelly, RRID:SCR_012091) in PBSuite 15.8.24 [19]. After gap closing, BESST 2.2.6 [20] and LINKS 1.8.5 [21] were used to perform scaffolding with Illumina and PacBio reads, respectively. Scaffolds were polished using Racon (commit version: 083444) [22] with PacBio long reads.

PacBio Iso-Seq reads were mapped onto scaffolds using GMAP (GMAP, RRID:SCR_008992) version 2017-08-15 [23], and then L_RNA_scaffolder [24] was used to concatenate scaffolds based on the results of Iso-Seq read mapping. Scaffolds were polished using Pilon 1.22 (Pilon, RRID:SCR_014731) [25] with PCR-free Illumina reads used for the MaSuRCA assembly. BUSCO 3.0.2 (Benchmarking Universal Single-Copy Orthologs, RRID:SCR_015008) [26] with a metazoan data set was used to evaluate the polished final genome assembly.

### Genome size estimation

PCR-free, paired-end reads used for genome assembly were analyzed. K-mers in the data set were counted with Jellyfish 2.2.3 (Jellyfish, RRID:SCR_005491) [27] (Supplementary Figure 1). The genome size of *P. naikaiensis* was estimated from obtained k-mer frequencies using GenomeScope web tools [28].

### Repeat analysis

Repetitive sequences in the assembled genome were identified using RepeatModeler 1.0.11 (RepeatModeler, RRID:SCR_015027) [29] and RepeatMasker 4.0.7 (RepeatMasker, RRID:SCR_012954) [30].

### Transcriptome assembly, gene prediction, and gene annotation

Adapter sequences and low-quality (<Q30) reads in the resulting RNA-seq paired-end data were removed using Trimmomatic (Trimmomatic, RRID:SCR_011848). Cleaned reads were assembled using Trinity 2.1.1 (Trinity, RRID:SCR_013048) [31] with default settings and the strand-specific option. In addition, genome-guided transcriptome assembly was performed. RNA-seq reads were mapped onto the genome using STAR 2.5.2a (STAR, RRID:SCR_015899) [32] and then mapped reads were assembled using Trinity. *De novo* assembled Illumina transcriptome and PacBio Iso-Seq sequences were mapped onto the genome using minimap-2 version 2.6 [33] with the “-ax splice” option. These mapping results and the genome-guided assembly of Illumina RNA-seq reads were integrated based on genome sequences using PASA 2.2.0 (PASA, RRID:SCR_014656) [34]. Putative full-length transcripts having both a 5′ and a 3′ untranslated region were detected using TransDecoder 5.0.2 [35]. These full-length transcripts were used as a training set for gene prediction. *De novo* transcriptome assembly of a data set containing 15 xenacoelomorphs (Supplementary Table 2) was also performed, following the procedure described above to create similarity hints for gene prediction. Assembled sequences of other acoels were translated into protein sequences using TransDecoder and then mapped against the *P. naikaiensis* genome using Exonerate 2.2.0 (Exonerate, RRID:SCR_016088) [36]. A final set of gene models reflecting hint information was generated with AUGUSTUS 3.2.1 (Augustus, RRID:SCR_008417) [37]. Gene models were annotated using BLAST searches (E-value cutoff of 10^−5^) against the NCBI RefSeq protein database release 88. Protein domains in gene models were detected using HMMER 3.1b2 (Hmmer, RRID:SCR_005305) [38] and Pfam-A 31.0 under default settings, except for an E-value cutoff of 10^−5^.

### A draft assembly

#### Mitochondrial genome

The complete, closed circular mitochondrial genome of *P. naikaiensis* was recovered from genome-sequencing data. The mitochondrial genome is 17,787 base-pairs long and contains 12 protein-coding genes, small and large ribosomal RNAs, and 22 predicted transfer RNAs (Supplementary Figure 2). When *cox1* was positioned at the start of the genome on the “positive” strand, 8 protein-coding genes were found in the same strand while *nad2, cytb*, and *nad5* were found on the “negative” strand (Supplementary Figure 2). Both ribosomal RNAs were found separately on the positive strand. Although the number of mitochondrial genes of *P. naikaiensis* is comparable to that of the previously reported mitochondrial genes of *Archaphanostoma ylvae* [8], the order of genes in the genomes was quite different between them.

#### Nuclear genome

K-mer analysis showed that the *P. naikaiensis* genome constitutes ∼654 Mb (Table 1; Supplementary Figure 1). Illumina paired-end and mate-pair, and PacBio reads provided 204x and 221x, and 73x coverage of the estimated genome, respectively (Supplementary Table 1 and Supplementary Figure 1). The assembly appeared to plateau during both scaffolding and contig formation (Supplementary Figure 3). As a result, the draft assembly comprised 656 Mb (Table   1), very close to the estimated genome size. The scaffold N50 reached 117 kb, and 12 scaffolds were >500 kb in length (Table 1; Supplementary Table 3). Inserted gaps made up only 1.7% of the total scaffold assembly (Supplementary Table 3). The contig N50 was 57 kb, and 41 contigs exceeded 250 kb (Table   1; Supplementary Table 3). The guanine + cytosine content of the genome was estimated at 39.1% (Table   1; Supplementary Figure 4).

**Table 1:** Genome assembly statistics for *P. naikaiensis*

Genome feature	Value
Estimated genome size*	654.1 Mb
Assembled genome size	656.1 Mb
Scaffolds (≥500 bp)	
No.	12,072
N50	117 kb
Contigs (≥500 bp)	
No.	24,071
N50	57 kb
Gaps	1.66%
Repetitive sequences	69.8%
Guanine + cytosine content	39.1%
Predicted protein-coding genes (loci)	22,143
Genes with transcript support	99%
Mean transcript length	2447 nucleotides
Mean exon frequency per gene	5.7
BUSCO analysis	
Complete	748/978 (76.5%)
Fragmented	37/978 (3.8%)

^*^Estimated by k-mer analysis of Illumina PCR-free reads as shown in Supplementary Figure 1.

Analysis of repetitive sequences showed that ∼69.8% of the genome consists of repetitive sequences (Tables 1 and 2; this value was estimated by deletion of overlapped sequences from the total data). DNA transposons (mutator-like transposable elements [MULEs], Marverick, hAT, and others), retrotransposons (long terminal repeats [LTRs], long and short interspersed nuclear elements), and other repetitive sequences (including low-complexity repeats and simple repeats) represented 12.2%, 41.5%, and 2.2% of the assembly, respectively (Table 2). The most prominent family was Gypsy of LTR, occupying 28.2% of the genome. In addition, the genome contains unclassified repetitive elements that accounted for 20.4% of it (Table 2). Thus, the *P. naikaiensis* genome contains a comparatively high percentage of repetitive sequences.

**Table 2:** Repetitive sequences in the *P. naikaiensis* genome assembly

Class	%
DNA transposons	12.2
MULE	5.7
Maverick	4.5
hAT	0.9
Others	1.1
Retrotransposons	41.5
LTR	35.7
Gypsy	28.2
Copia	1.5
Pao	1.1
Others	4.9
Long interspersed nuclear elements	4.8
CR1	1.7
CRE	1.0
L2	0.9
Others	1.2
Short interspersed nuclear elements	1.0
Others	2.2
RNA	0.03
Low complexity	0.07
Satellite	0.4
Simple repeat	1.7
Unclassified	20.4
Total (excluding overlapped sequences)	69.8

An interesting question concerns the locations of transposable elements (TEs) in introns. We found that 32,110 TEs are present in intron regions; 29%, 28%, and 12% of them correspond to “uncharacterized,” “LTR (Gypsy),” and “DNA transposon (MULE),” respectively. On the other hand, we failed to find introns that are composed of only TEs.

### Transcriptomes

Transcriptome data, especially those from PacBio Iso-Seq long reads, provided a set of high-quality RNA data (Supplementary Table 1). The mean length of transcriptomes was 2,447 nucleotides, and the mean number of exons per gene was 5.7 (Table 1).

### Gene modeling

Gene modeling of the *P. naikaiensis* genome produced 22,143 protein-coding genes (Table 1). As mentioned above, we obtained a set of high-quality RNA data. As a result, 99% of gene models were substantiated by the transcriptomes (Table 1).

BUSCO analysis indicated that 76.5% and 3.8% of them were supported as complete and fragmented genes, respectively (Table 1).

### Gene annotation

Gene families predicted by RefSeq (BLAST), Pfam (HMMER), and PANTHER (HMMER) were 15,294, 13,225, and 17,384 in number, respectively (Supplementary Figure 5). Using Pfam-supported families, we examined the number of gene families. Table   3 shows numbers of putative transcription regulator genes in the *P. naikaiensis* genome. The 2 most abundant families were zinc finger (C2H2 type) and homeobox domain-containing genes, with 73 and 62 members, respectively. Twenty each were annotated to the helix-loop-helix and zinc finger (C4 types) families. Although more detailed analysis is required, the *P. naikaiensis* genome appears to contain numbers of transcription regulator genes comparable to those of other bilaterian genomes.

**Table 3:** Numbers of putative transcriptional regulator genes in the *P. naikaiensis* genome

Accession	ID	Description	No. of genes
PF00010	HLH	Helix-loop-helix DNA-binding domain	20
PF00046	Homeobox	Homeobox domain	62
PF00096	zf-C2H2	Zinc finger, C2H2 type	73
PF00104	Hormone_recep	Ligand-binding domain of nuclear hormone	14
PF00105	zf-C4	Zinc finger, C4 type	20
PF00157	Pou	Pou domain	3
PF00170	bZIP_1	bZIP transcription factor	13
PF00178	Ets	Ets domain	13
PF00250	Fork_head	Fork head domain	11
PF00292	PAX	“Paired box” domain	5
PF00319	SRF-TF	SRF-type transcription factor	2
PF00320	GATA	GATA zinc finger	7
PF00505	HMG_box	HMG (high mobility group) box	12
PF00554	RHD	Rel homology domain (RHD)	1
PF00853	Runt	Runt domain	1
PF00870	P53	P53 DNA-binding domain	1
PF00907	T-box	T-box	4
PF01388	ARID	ARID/BRIGHT DNA-binding domain	4
PF01530	zf-C2HC	Zinc finger, C2HC type	2
PF02376	CUT	CUT domain	3
PF03299	TF_AP-2	Transcription factor AP-2	1
PF05044	Prox1	Homeo-prospero domain	1
PF07527	Hairy_orange	Hairy orange	1
PF07716	bZIP_2	Basic region leucine zipper	11

A similar analysis was carried out on putative signaling molecule genes (Table 4). The largest gene family was tyrosine kinase, represented by 316 genes. In addition, epidermal growth factor (EGF)-like domain genes, G-protein α subunit genes, and regulator of G-protein signaling genes numbered 28, 31, and 16, respectively (Table 4).

**Table 4:** Numbers of genes encoding putative signaling molecules in the *P. naikaiensis* genome

Accession	ID	Description	No. of genes
PF00008	EGF	EGF-like domain	28
PF00019	TGF_beta	Transforming growth factor β like	5
PF00110	wnt	wnt family	4
PF00167	FGF	Fibroblast growth factor	3
PF00219	IGFBP	Insulin-like growth factor binding protein	1
PF00503	G-alpha	G-protein α subunit	31
PF00615	RGS	Regulator of G protein signaling	16
PF00631	G-gamma	GGL domain	5
PF00688	TGFb_propeptide	Transforming growth factor β propeptide	3
PF00778	DIX	DIX domain	5
PF01017	STAT_alpha	STAT protein, all-α domain	2
PF01534	Frizzled	Frizzled/Smoothened family membrane region	6
PF02262	Cbl_N	CBL proto-oncogene N-terminal domain 1	2
PF02377	Dishevelled	Dishevelled specific domain	1
PF02761	Cbl_N2	CBL proto-oncogene N-terminus, EF hand-like	2
PF02762	Cbl_N3	CBL proto-oncogene N-terminus, SH2-like domain	2
PF02864	STAT_bind	STAT protein, DNA binding domain	2
PF02865	STAT_int	STAT protein, protein interaction domain	2
PF07714	Pkinase_Tyr	Tyrosine kinase	316

### Genome browser

**Figure 2:**
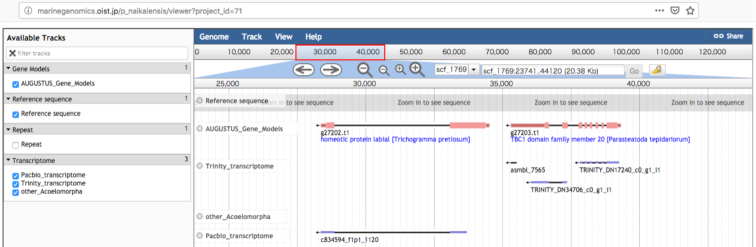
A screen shot of the genome browser of *Praesagittifera naikaiensis*[40].

A genome browser was established for the assembled sequences using the JBrowser 1.12.3 [39]. Its URL is [40] (Fig. 2). The gene annotations from the protein domain search and BLAST search have similarly been shown on the site.

## Availability of supporting data and materials

Genomic and transcriptomic sequence reads have been deposited in the DDBJ sequence read archive under accession No. PRJDB7329. All data are also available from the *GigaScience* GigaDB repository [41].

## Additional files

**Supplementary Table 1:** Sequence data summary.

**Supplementary Figure 1:** K-mer analysis and genome size estimation of *Praesagittifera naikaiensis* genomic DNA reads.

**Supplementary Table 2:** Xenacoelomorph dataset used for gene prediction.

**Supplementary Figure 2:** A preliminary circular assembly of the mitochondria genome of *Praesagittifera naikaiensis*.

**Supplementary Figure 3:** Accumulation of assembled sequences (contigs, blue and scaffolds, red) reaching over 600 Mb.

**Supplementary Table 3:** Summary of the *Praesagittifera naikaiensis* genome assembly.

**Supplementary Figure 4:** GC content of the *Praesagittifera naikaiensis* genome.

**Supplementary Figure 5:** *Praesagittifera naikaiensis* gene annotation.

## Abbreviations

BUSCO: Benchmarking Universal Single-Copy Orthologs; EGF: epidermal growth factor; kb: kilobase pair; LTR: long terminal repeat; Mb: megabase pair; MULE: mutator-like transposable element; PCR: polymerase chain reaction; RNA-seq: RNA sequencing; TE: transposable element.

## Competing interests

The authors declare that they have no competing interests.

## Funding

This work was funded by Okinawa Institute of Science and Technology Internal Funds to the Marine Genomics Unit (N.S.). This work was also supported by a JSPS grant (No. 17K07535) and a research grant from the Research Institute of Marine Invertebrates (Japan) to A.H.

## Author contributions

N.S., T.H.K., A.H., K.T., A.A., M.A.Y., and T.U. conceived and supervised the project. T.H.K. and T.I. collected the majority of samples. M.K. and A.A. performed sequencing. A.A., K.H., E.S., and T.H.K. performed analyses. N.S., A.A., and T.H.K. prepared the manuscript, and all authors approved the final manuscript.

## Supplementary Material

GIGA-D-18-00363_Original_Submission.pdf

GIGA-D-18-00363_Revision_1.pdf

Response_to_Reviewer_Comments_Original_Submission.pdf

Reviewer_1_Report_Original_Submission -- Bruce A Rosa 11/10/2018 Reviewed

Reviewer_2_Report_Original_Submission -- Mahul Chakraborty 11/22/2018 Reviewed

Reviewer_2_Report_Revision_1 -- Mahul Chakraborty 1/29/2019 Reviewed

Supplemental Files
