# Supplementary material for: A draft nuclear-genome assembly of the acoel flatworm Praesagittifera naikaiensis
Source: Gigascience. 2019 Apr 6;8(4):giz023. doi: 10.1093/gigascience/giz023 (PMC6451197; doi:10.1093/gigascience/giz023)
Supplement: GIGA-D-18-00363_Original_Submission.pdf [file giz023_giga-d-18-00363_original_submission.pdf]

GigaScience
A draft genome assembly of the acoel flatworm Praesagittifera naikaiensis
--Manuscript Draft--

**Manuscript Number:**	GIGA-D-18-00363
**Full Title:**	A draft genome assembly of the acoel flatworm Praesagittifera naikaiensis
**Article Type:**	Data Note
**Funding Information:**	Okinawa Institute of Science and Technology Graduate University (Internal Fund) — Dr. Noriyuki Satoh<br>Japan Society for the Promotion of Science (17K07535) — Dr. Akira Hikosaka
**Abstract:**	Background<br><br>Acoels are primitive bilaterians with very simple soft-bodies, in which many organs, including the gut, are undeveloped. Therefore they provide platforms for studying molecular and developmental mechanisms involved in the formation of the basic bilaterian body-plan, analyses of whole-body regeneration, and research on symbiosis with photosynthetic microalgae. Because genomic information is essential for future research on acoel biology, we sequenced and assembled the genome of an acoel, Praesagittifera naikaiensis.<br><br>Findings<br><br>The acoel genome size was estimated to be approximately 654 Mbp. More than 290x sequencing coverage was achieved using a combination of Illumina (paired end and mate-pair) and PacBio sequencing. This yielded a draft assembly of ~656-Mbp, with a scaffold N50 of 117 kb and contig N50 of 57 kb. RNA-seq and Iso-Seq data of embryos, larvae, and adults were also obtained. Although ~70% of the assembled sequences were comprised of repetitive sequences, including DNA transposons and retrotransposons, the draft genome was estimated to contain 22,143 protein-coding genes. BUSCO analyses supported completeness of 77% of the annotated genes, and 99% of the gene models were substantiated by transcripts. Pfam domain analyses provided a basic set of gene families for transcription factors and signaling molecules in this animal.<br><br>Conclusions<br><br>Our present sequencing and assembly of the P. naikaiensis genome are comparable to those of other metazoan genomes, providing basic information for future studies of genic and genomic attributes of this animal group. Such analyses may shed light on the origins and evolution of these simple bilaterians and on symbiosis with photosynthetic algae.
**Corresponding Author:**	Asuka Arimoto<br><br>JAPAN
**Corresponding Author Secondary Information:**	
**Corresponding Author's Institution:**	
**Corresponding Author's Secondary Institution:**	
**First Author:**	Asuka Arimoto
**First Author Secondary Information:**	
**Order of Authors:**	Asuka Arimoto<br>Tomoe Hikosaka-Katayama

Powered by Editorial Manager® and ProduXion Manager® from Aries Systems Corporation

	Akira Hikosaka
	Kunifumi Tagawa
	Toyoshige Inoue
	Tatsuya Ueki
	Masa-aki Yoshida
	Miyuki Kanda
	Eiichi Shoguchi
	Kanako Hisata
	Noriyuki Satoh
**Order of Authors Secondary Information:**	
**Additional Information:**	
**Question**	**Response**
Are you submitting this manuscript to a special series or article collection?	No
**Experimental design and statistics**<br><br>Full details of the experimental design and statistical methods used should be given in the Methods section, as detailed in our Minimum Standards Reporting Checklist. Information essential to interpreting the data presented should be made available in the figure legends.<br><br>Have you included all the information requested in your manuscript?	Yes
**Resources**<br><br>A description of all resources used, including antibodies, cell lines, animals and software tools, with enough information to allow them to be uniquely identified, should be included in the Methods section. Authors are strongly encouraged to cite Research Resource Identifiers (RRIDs) for antibodies, model organisms and tools, where possible.<br><br>Have you included the information requested as detailed in our Minimum Standards Reporting Checklist?	Yes

**Availability of data and materials**<br><br>All datasets and code on which the conclusions of the paper rely must be either included in your submission or deposited in publicly available repositories (where available and ethically appropriate), referencing such data using a unique identifier in the references and in the "Availability of Data and Materials" section of your manuscript.<br><br>Have you have met the above requirement as detailed in our Minimum Standards Reporting Checklist?	Yes

Powered by Editorial Manager® and ProduXion Manager® from Aries Systems Corporation

Manuscript

Click here to download Manuscript Satoh_Pnaikai_Genome_MS.180918.docx

GigaScience: DATA NOTE

A draft genome assembly of the acoel flatworm *Praesagittifera naikaiensis*

Asuka Arimoto[1+*], Tomoe Hikosaka-Katayama[2+], Akira Hikosaka[3], Kunifumi Tagawa[4], Toyoshige Inoue[4], Tatsuya Ueki[4,5], Masa-aki Yoshida[6], Miyuki Kanda[7], Eiichi Shoguchi[1], Kanako Hisata[1] and Noriyuki Satoh[1*]

[1]Marine Genomics Unit, Okinawa Institute of Science and Technology Graduate University, Onna, Okinawa 904-0495, Japan

[2]Natural Science Center for Basic Research and Development Center for Gene Science Division, Hiroshima University, Higashi-Hiroshima, Hiroshima 739-8527, Japan

[3]Division of Human Sciences, Graduate School of Integrated Arts and Sciences, Hiroshima University, Higashi-Hiroshima, Hiroshima 739-8521, Japan

[4]Marine Biological Laboratory, Graduate School of Science, Hiroshima University, Onomichi, Hiroshima 722-0073, Japan

[5]Department of Biological Science, Graduate School of Science, Hiroshima University, Higashi Hiroshima, Hiroshima 739-8526, Japan

[6]Marine Biological Science Section, Education and Research Center for Biological Resources, Faculty of Life and Environmental Science, Shimane University, Kamo 194, Okinoshima-cho, Oki, Shimane, 685-0024, Japan

[7]DNA Sequence Section, Okinawa Institute of Science and Technology Graduate University, Onna, Okinawa 904-0495, Japan

[+] These authors contributed equally

*Corresponding authors: Asuka Arimoto (asuka.arimoto@oist.jp), Noriyuki Satoh (norisky@oist.jp)

Abstract

Background: Acoels are primitive bilaterians with very simple soft-bodies, in which many organs, including the gut, are undeveloped. Therefore they provide platforms for studying molecular and developmental mechanisms involved in the formation of the basic bilaterian body-plan, analyses of whole-body regeneration, and research on symbiosis with photosynthetic microalgae. Because genomic information is essential for future research on acoel biology, we sequenced and assembled the genome of an acoel, *Praesagittifera naikaiensis*.

Findings: The acoel genome size was estimated to be approximately 654 Mbp. More than 290x sequencing coverage was achieved using a combination of Illumina (paired end and mate-pair) and PacBio sequencing. This yielded a draft assembly of ~656-Mbp, with a scaffold N50 of 117 kb and contig N50 of 57 kb. RNA-seq and Iso-Seq data of embryos, larvae, and adults were also obtained. Although ~70% of the assembled sequences were comprised of repetitive sequences, including DNA transposons and retrotransposons, the draft genome was estimated to contain 22,143 protein-coding genes. BUSCO analyses supported completeness of 77% of the annotated genes, and 99% of the gene models were substantiated by transcripts. Pfam domain analyses

provided a basic set of gene families for transcription factors and signaling molecules in this animal.

Conclusions: Our present sequencing and assembly of the *P. naikaiensis* genome are comparable to those of other metazoan genomes, providing basic information for future studies of genic and genomic attributes of this animal group. Such analyses may shed light on the origins and evolution of these simple bilaterians and on symbiosis with photosynthetic algae.

Key words: Acoel, *Praesagittifera naikaiensis*, Draft genome, ~22,000 Predicted genes

Data description

Background

Acoels are small, very simple, planula-like animals lacking a coelom, a gut, and a central nervous system. Traditional taxonomy positioned the Acoela as the most basal order of the phylum Platyhelminthes [1]. Recent studies, based on molecular and genomic data, however, have suggested that acoels are members of a new phylum, the Xenacoelomorpha, together with nemertodermatids and xenoturbellids [2, 3, 4]. Whether xenacoelomorphs are basal to all other bilaterians or whether they have close affinity to ambulacrarians is a matter of debate. Nonetheless, acoels are pivotal to understanding the origins and evolution of bilaterians. Acoels also provide a platform for molecular studies of whole-body regeneration [5] and symbiosis with photosynthetic microalgae.

Sampling and sequencing

Biological materials

The marine acoel worm, *Praesagittifera naikaiensis*, is 2-3 mm in length (Fig. 1A) [6]. These worms are easily found at seashores of the Seto Inland Sea, especially during the early summer season (Fig. 1B). Adults contains many symbiotic microalgae, *Tetraselmis* sp. (Fig. 1C), which are integrated during juvenile growth (Fig. 1D). Adults

were collected at the seashore near the Marine Biological Laboratory of Hiroshima University and maintained in aquaria in the laboratory on a 12-h light/12-h dark photoperiod. Naturally laid eggs were collected and cultured for embryogenesis (Fig. 1D). After washing the embryos with filtered seawater, genomic DNA was extracted from them using the phenol/chloroform extraction. Adults were also prepared for sequencing separately. Total RNA extraction was performed using TRIzol Reagent (Invitrogen, 15596-026) and RNeasy mini Kit (Qiagen, 74104).

Library preparation and sequencing

All sequencing libraries were constructed according to the manufacturers' standard protocols. Briefly, PCR-free, paired-end libraries were prepared using an Illumina TruSeq DNA PCR-Free LT Library Prep Kit (Illumina, FC-121-3001). Four mate-pair libraries were prepared using a Nextera Mate Pair Library Prep Kit (Illumina, FC-132-1001) (Additional file 1). An RNA-seq library was prepared using a TruSeq Stranded mRNA LT Sample Prep Kit (Illumina, RS-122-2101). These libraries were sequenced using the Illumina HiSeq 2500 platform (Additional file 1).

A PacBio DNA library was prepared according to the manufacturers' 10-kb template preparation protocol. cDNAs for Iso-Seq libraries were prepared using a SMARTer PCR cDNA synthesis kit (Clontech, 634925). The SageELF size selection system (Sage Science, MA, USA) was used following the manufacturers' standard

protocol (Additional file 1). A SMRTbell Templete Prep Kit 1.0 (Pacific Biosciences, 100-259-100) was used for both types of PacBio library preparation. Libraries were sequenced using a PacBio RSII sequencer employing P6-C4 chemistry (Pacific Biosciences, 100-372-700) with 360 min movie lengths. A total of 52 and 8 SMRT Cells were sequenced for long-read DNA and Iso-Seq RNA libraries, respectively.

Nuclear genome assembly

Adapter sequences in PCR-free and mate-pair Illumina reads were removed with Trimmomatic 0.36 [7] and NextClip 1.3.1 [8], respectively. Low quality (<Q20) insert regions were removed using Sickle 1.33 [9] after adapter cleanup. Reads that lacked a corresponding pair were discarded. Trimmed reads were mapped onto the *P. naikaiensis* mitochondrial genome using BWA 0.7.12 [10], while read pairs that mapped onto the mitochondrial genome were excluded from the dataset. PacBio long-reads were also mapped against the mitochondrial genome using BLASR (commit version: 5.3.574e1c2) [11]. Unmapped or cleaned-up Illumina and PacBio reads were assembled using the MaSuRCA assembler 3.2.2 [12].

Putative heterozygous and/or polymorphic sequences that remained in the assembled genome were merged as homozygous sequences using redundans 0.13c [13]. Gaps in the homozygous genome were filled using PBJelly in PBSuite 15.8.24 [14]. After gap closing, BESST 2.2.6 [15] and LINKS 1.8.5 [16] were used to perform

scaffolding with Illumina and PacBio reads, respectively. Scaffolds were polished using Racon (commit version: 083444) [17] with PacBio long reads.

PacBio Iso-Seq reads were mapped onto scaffolds using GMAP version 2017-08-15 [18], and then L_RNA_scaffolder [19] was used to concatenate scaffolds based on the results of Iso-Seq read mapping. Scaffolds were polished using Pilon 1.22 [20] with PCR-free Illumina reads used for MaSuRCA assembly. BUSCO 3.0.2 [21] with a metazoan dataset and CEGMA 2.5 [22] were used to evaluate the polished final genome assembly.

Genome size estimation

PCR-free paired-end reads used for genome assembly were analyzed. K-mers in the dataset were counted with Jellyfish 2.2.3 [23] (Additional file 4). The genome size of *P. naikaiensis* was estimated from obtained k-mer frequencies using GenomeScope web tools [24].

Repeat analysis

Repetitive sequences in the assembled genome were identified using RepeatModeler 1.0.11 [25] and RepeatMasker 4.0.7 [26]. The Kimura substitution level of each transposable sequence was calculated using scripts in the RepeatMasker package.

Transcriptome assembly, gene prediction and gene annotation

Adapter sequences and low quality (<Q30) reads in the obtained RNA-seq paired-end data were removed using Trimmomatic. Cleaned-up reads were assembled using Trinity 2.1.1 [27] with default settings, except the strand-specific option. Parallel, genome-guided transcriptome assembly was performed. RNA-seq reads were mapped onto the genome using STAR 2.5.2a [28] and then mapped reads were assembled using Trinity. *De novo* assembled Illumina transcriptome and PacBio Iso-Seq sequences were mapped onto the genome using minimap-2 version 2.6 [29] with the "-ax splice" option. These mapping results and the genome guided assembly of Illumina RNA-seq reads were integrated based on genome sequences using PASA 2.2.0 [30]. Putative full-length (FL) transcripts having both a 5' UTR and a 3' UTR were detected using TransDecoder 5.0.2 [31]. These FL transcripts were used as a training set for gene prediction. *De novo* transcriptome assembly of a dataset containing 15 xenacoelomorphs (Additional file 3) was also performed following the procedure described above to create similarity hints for gene prediction. Assembled sequences of other acoels were translated into protein sequences using TransDecoder and then mapped against the *P. naikaiensis* genome using Exonerate 2.2.0 [32]. A final set of gene models reflecting hint information was generated with AUGUSTUS 3.2.1 [33]. Gene models were annotated using BLAST searches (E-value cutoff of 10^{-5}) against the NCBI RefSeq protein database release 88. Protein domains in gene models were detected using HMMER 3.1b2 [34] and Pfam-A

31.0 under default settings, except for an E-value cutoff of 10^{-5}. A genome browser integrating these annotation data is available at http://marinegenomics.oist.jp/gallery/.

A draft assembly

K-mer analysis showed that the *P. naikaiensis* genome was approximately 654 Mb in size (Table 1; Additional file 4). Paired-end, mate-pair, and PacBio reads provided 204x, 221x, and 73x coverage of the estimated genome, respectively (Additional files 1 and 2). The assembly appeared to reach plateau during both scaffolding and contig formation (Additional file 2). As a result, the draft assembly comprised a total 656 Mb (Table 1), very close to the estimated genome size. Scaffold N50 reached 117 kb, and 12 scaffolds were over 500 kb in length (Table 1; Additional file 2). Inserted gaps were only 1.7% of the total scaffold assembly (Additional file 2). Contig N50 was 57 kb, and 41 contigs exceeded 250 kb (Table 1; Additional file 2). The GC content of the genome was estimated 39.1% (Table 1; Additional file 5).

Analysis of repetitive sequences showed that approximately 68% of the genome consists of repetitive sequences (Table 1 and Table 2). DNA transposons, retrotransposons, and others represented 11.3%, 33.8%, and 19% of the assembly, respectively. The most prominent family was Gypsy of LTR (long terminal repeat), occupying 27.0% of the genome.

Gene modeling

Gene modeling of the *P. naikaiensis* genome produced 22,143 protein-coding genes (Table 1). BUSCO analysis indicated that 68% and 8% of them were supported as complete single-copy and double-copy genes, respectively (Table 1). On the other hand, CEGMA analysis demonstrated that 82% and 90% of them were supported by completed and partial genes (Table 1).

Transcriptome data, especially those from PacBio Iso-Seq long-reads, provided a set of high-quality RNA data (Additional file 1). As a result, 99% of gene models were substantiated by transcriptomes (Table 1). An average length of transcriptomes was 2,447 nucleotides, and an average number of exons per gene was 5.7 (Table 1).

Gene annotation

Gene families predicted by RefSeq (BLAST), Pfam (HMMER) and PANTHER (HMMER) were 15,294, 13,225 and 17,384 in number, respectively (Additional file 7). Using Pfam (HMMER)-supported families, we examined the number of gene families. Table 3 shows numbers of putative transcription regulator genes in the *P. naikaiensis* genome. The two most abundant families were Zinc-finger (C2H2 type) and homeobox domain-containing genes, with 73 and 62 members, respectively. Twenty each were annotated to HLH and Zinc-finger (C4 types) families. Although more detailed analysis

is required, the *P. naikaiensis* genome appears to contain transcription regulator genes comparable to those of other bilaterian genomes.

A similar analysis was carried out on putative signaling molecule genes (Table 4). The largest gene family was tyrosine kinase, represented by 316 genes. In addition, EGF-like domain genes, G-protein alpha subunit genes, and Regulator of G-protein signaling genes numbered 28, 31, and 16, respectively (Table 4).

Availability of data

Genomic and transcriptomic sequence reads have been deposited in the DDBJ sequence read archives under accession number PRJDB7329. A genome browser is accessible at: http://marinegenomics.oist.jp/gallery/.

Additional files

Additional file 1: Supplementary Table 1: Sequence data summary.

Additional file 2: Supplementary Table 2: Summary of the Praesagittifera naikaiensis genome assembly.

Additional file 3: Supplementary Table 3: Xenacoelomorpha dataset used for gene prediction.

Additional file 4: Supplementary Figure 1: Summary of *Praesagittifera naikaiensis* genomic DNA reads.

Additional file 5: Supplementary Figure 2: Accumulation of sequence reads (contigs, blue and scaffolds, red) reaching over 600 Mbp.

Additional file 6: Supplementary Figure 3: GC content of *Praesagittifera naikaiensis* genome.

Additional file 7: Supplementary Figure 4: *Praesagittifera naikaiensis* gene annotation.

Competing interests

The authors declare that they have no competing interests.

Authors' contributions

NS, THK, AH, KT, AA, MAY, and TU conceived and supervised the project. THK and TI collected the majority of samples. MK and AA performed sequencing. AA, KH, ES and THK performed analyses. NS, AA, and THK prepared the manuscript and all authors approved the final manuscript.

Acknowledgments

The work was funded by OIST Internal Fund to Marine Genomics Unit (NS). This work was also supported by JSPS grant (No.17K07535) and a research grant from the

Research Institute of Marine Invertebrates to AH. We are grateful to Dr. Steven D. Aird for his technical editing of the manuscript.

References

1. Hyman LH. The Invertebrates: Platyhelminthes and Rhynchocoela; the Acoelomate Bilateria. New York: McGraw-Hill; 1951.
2. Ruiz-Trillo I, Riutort M, Littlewood DTJ, Hejnol EA, Baguñà J. Acoel flatworms: earliest extant bilaterian metazoans, not members of Platyhelminthes. *Science* 1999;283:1919-23.
3. Philippe H, Brinkmann H, Copley RR, Moroz LL, Nakano H, Poustka AJ, et al. Acoelomorph flatworms are deuterostomes related to *Xenoturbella*. *Nature* 2011;470:255-8.
4. Cannon JT, Vellutini BC, Smith J 3rd, Ronquist F, Jondelius U, Hejnol A. Xenacoelomorpha is the sister group to Nephrozoa. *Nature* 2016;530:89-93.
5. Srivastava M, Mazza-Curll KL, van Wolfswinkel JC, Reddien PW. Whole-body acoel regeneration is controlled by Wnt and Bmp-Admp signaling. *Curr Biol*. 2014;24:1107-13.
6. Hikosaka-Katayama T, Hikosaka A. Artificial Rearing System for *Praesagittifera naikaiensis* (Acoela, Acoelomorpha). *Studies in Human Science* 2015;10:17-23.

7. Bolger AM, Lohse M, Usadel B. Trimmomatic: a flexible trimmer for Illumina sequence data. *Bioinformatics* 2014;30:2114-20.
8. Leggett RM, Clavijo BJ, Clissold L, Clark MD, Caccamo M. NextClip: an analysis and read preparation tool for Nextera Long Mate Pair libraries. *Bioinformatics* 2014; 30:566-8.
9. Sickle https://github.com/najoshi/sickle
10. Li H. Aligning sequence reads, clone sequences and assembly contigs with BWA-MEM. 2013. arXiv:1303.3997v2 [q-bio.GN].
11. Chaisson MJ. Tesler G. Mapping single molecule sequencing reads using basic local alignment with successive refinement (BLASR): application and theory. *BMC Bioinformatics* 2012;13:238.
12. Zimin AV, Marçais G, Puiu D, Roberts M, Salzberg SL, Yorke JA. The MaSuRCA genome assembler. *Bioinformatics* 2013;29:2669-77.
13. Pryszcz LP, Gabaldón T. Redundans: an assembly pipeline for highly heterozygous genomes. *Nucleic Acids Res*. 2016;44:e113.
14. English AC, Richards S, Han Y, Wang M, Vee V, Qu J, et al. Mind the gap: upgrading genomes with Pacific Biosciences RS long-read sequencing technology. *PLoS One* 2012;7:e47768.
15. Sahlin K, Chikhi R, Arvestad L. Assembly scaffolding with PE-contaminated mate-pair libraries. *Bioinformatics* 2016;32:1925-32.

16. Warren RL, Yang C, Vandervalk BP, Behsaz B, Lagman A, Jones SJ, et al. LINKS: Scalable, alignment-free scaffolding of draft genomes with long reads. *Gigascience* 2015;4:35.

17. Vaser R, Sović I, Nagarajan N, Šikić M. Fast and accurate de novo genome assembly from long uncorrected reads. *Genome Res*. 2017;27:737-46.

18. Wu TD, Watanabe CK. GMAP: a genomic mapping and alignment program for mRNA and EST sequences. *Bioinformatics* 2005;21:1859-75.

19. Xue W, Li JT, Zhu YP, Hou GY, Kong XF, Kuang YY, et al. L_RNA_scaffolder: scaffolding genomes with transcripts. *BMC Genomics*. 2013;14:604.

20. Walker BJ, Abeel T, Shea T, Priest M, Abouelliel A, Sakthikumar S, et al. Pilon: an integrated tool for comprehensive microbial variant detection and genome assembly improvement. *PLoS One* 2014;9:e112963.

21. Waterhouse RM, Seppey M, Simão FA, Manni M, Ioannidis P, Klioutchnikov G, et al. BUSCO applications from quality assessments to gene prediction and phylogenomics. *Mol Biol Evol*. 2018;35:543-8.

22. Parra G, Bradnam K, Korf I. CEGMA: a pipeline to accurately annotate core genes in eukaryotic genomes. *Bioinformatics* 2007;23:1061-7.

23. Marçais G, Kingsford C. A fast, lock-free approach for efficient parallel counting of occurrences of k-mers. *Bioinformatics* 2011;27:764-70.

24. Vurture GW, Sedlazeck FJ, Nattestad M, Underwood CJ, Fang H, Gurtowski J, et al. GenomeScope: fast reference-free genome profiling from short reads. *Bioinformatics* 2017;33:2202-4.

25. RepeatModeler http://www.repeatmasker.org/RepeatModeler

26. RepeatMasker http://www.repeatmasker.org

27. Grabherr MG, Haas BJ, Yassour M, Levin JZ, Thompson DA, Amit I, et al. Full-length transcriptome assembly from RNA-Seq data without a reference genome. *Nat Biotechnol*. 2011;29:644-52.

28. Dobin A, Davis CA, Schlesinger F, Drenkow J, Zaleski C, Jha S, et al. STAR: ultrafast universal RNA-seq aligner. *Bioinformatics* 2013;29:15-21.

29. Li H. Minimap2: pairwise alignment for nucleotide sequences. *Bioinformatics* 2018. doi: 10.1093/bioinformatics/bty191.

30. Haas BJ, Delcher AL, Mount SM, Wortman JR, Smith RK Jr, Hannick LI, et al. Improving the *Arabidopsis* genome annotation using maximal transcript alignment assemblies. *Nucleic Acids Res*. 2003;31:5654-66.

31. TransDecoder https://github.com/TransDecoder/TransDecoder

32. Slater GS, Birney E. Automated generation of heuristics for biological sequence comparison. *BMC Bioinformatics* 2005;6:31.

33. Stanke M, Diekhans M, Baertsch R, Haussler D. Using native and syntenically mapped cDNA alignments to improve de novo gene finding. *Bioinformatics* 2008;24:637-44.

34. HMMER http://hmmer.org

Figure legends

Figure 1: The acoel worm, *Praesagittifera naikaiensis.* (A) An adult, dorsal view. Anterior, top and posterior, bottom. Green dots throughout the entire body are symbiotic green algae. Two eggs are seen in the center of the worm. (B) An enormous number of adults gathering on the sandy seashore, resembling dark masses. (C) A peripheral region of an adult worm showing symbiotic microalgae, *Tetraselmis* sp. (D) Embryogenesis. Newly laid egg within the eggshell (i), four-cell stage embryo (ii), gastrula (iii), flattened-stage embryo (iv), newly-hatched aposymbiotic algae (v), and symbiotic juvenile with symbiotic algae (vi). Scale = 50 µm in (A), (C), (D); 10 cm in (B).

Supplementary Figure 1: Summary of *Praesagittifera naikaiensis* genomic DNA reads. X-axis shows coverage and Y-axis frequency.

Supplementary Figure 2: Accumulation of sequence reads (contigs, blue and scaffolds, red) reaching over 600 Mbp. X-axis shows sequence index and Y-axis cumulative length (Mbp).

Supplementary Figure 3: GC content of *Praesagittifera naikaiensis* genome. X-axis shows ratios of GC content (%) and Y-axis number of reads in 500-bp window.

Supplementary Figure 4: *Praesagittifera naikaiensis* gene annotation. A total of 22,143 genes were annotated by different databases.

Table 1: Genome assembly statistics

Genome features	*Praesagittifera naikaiensis*
Estimated genome size*	654.1 Mb
Assembled genome size	656.1 Mb
Scaffolds (≥500 bp)	
Number	12,072
N50	117 kb
L50	1,648
Contigs (≥500 bp)	
Number	24,071
N50	57 kb
L50	3279
Gaps	1.66%
GC content	39.1%
Repetitive sequences	67.94%
BUSCO analysis	
Complete and single-copy	68.1%
Complete and duplicated	8.4%
Fragmented	3.8%
Missing	19.7%
CEGMA analysis	
Complete	81.85%
Partial	90.32%
Predicted protein-coding genes (loci)	22,143
Genes with transcript support	99%
Mean transcript length	2447 nt
Mean exon frequency per gene	5.7

* Estimated by k-mer analysis of Illumina PCR-free reads as shown Supplementary Figure 1.

Table 2: Repetitive sequences in the *Praesagittifera naikaiensis* genome

Class			Percentage in the assembly
DNA transposons			11.25
		MULE	4.52
		Maverick	4.42
		hAT	1.64
Retrotransposons			
	LTR		33.79
		Gypsy	27.04
		Copia	1.4
		Pao	1.14
	LINE		3.34
		CR1	1.23
		L2	0.91
	SINE		0.53
Others			
	Satellite		0.42
	Simple repeat		0.31
	Unknown		18.3
Total			67.94

Table 3: Numbers of putative transcriptional regulator genes in the *Praesagittifera naikaiensis* genome

Accession	Description	No. of genes
PF00010	Helix-loop-helix DNA-binding domain	20
PF00046	Homeobox domain	62
PF00096	Zinc finger, C2H2 type	73
PF00104	Ligand-binding domain of nuclear hormone	14
PF00105	Zinc finger, C4 type	20
PF00157	Pou domain	3
PF00170	bZIP transcription factor	13
PF00178	Ets-domain	13
PF00250	Fork head domain	11
PF00292	'Paired box' domain	5
PF00319	SRF-type transcription factor	2
PF00320	GATA zinc finger	7
PF00505	HMG (high mobility group) box	12
PF00554	Rel homology domain (RHD)	1
PF00751	DM DNA binding domain	0
PF00853	Runt domain	1
PF00870	P53 DNA-binding domain	1
PF00907	T-box	4
PF01388	ARID/BRIGHT DNA-binding domain	4
PF01530	Zinc finger, C2HC type	2
PF01586	Myogenic Basic domain	0
PF02023	SCAN domain	0
PF02178	AT hook motif	0
PF02376	CUT domain	3
PF03299	Transcription factor AP-2	1
PF03529	Otx1 transcription factor	0
PF03615	GCM motif protein	0
PF03826	OAR domain	0
PF05044	Homeo-prospero domain	1
PF06621	Single-minded protein C-terminus	0
PF07527	Hairy Orange	1
PF07710	P53 tetramerisation motif	0
PF07716	Basic region leucine zipper	11
PF12598	T-box transcription factor	0

Table 4: Numbers of genes encoding putative signaling molecules in the *Praesagittifera naikaiensis* genome

Accession	Description	No. of genes
PF00008	EGF-like domain	28
PF00015	Methyl-accepting chemotaxis protein (MCP) signalling	0
PF00019	Transforming growth factor beta like	5
PF00049	Insulin/IGF/Relaxin family	0
PF00110	wnt family	4
PF00167	Fibroblast growth factor	3
PF00219	Insulin-like growth factor binding protein	1
PF00341	PDGF/VEGF domain	0
PF00503	G-protein alpha subunit	31
PF00615	Regulator of G protein signaling	16
PF00631	GGL domain	5
PF00672	HAMP domain	0
PF00688	TGF-beta propeptide	3
PF00778	DIX domain	5
PF01017	STAT protein, all-alpha domain	2
PF01091	PTN/MK heparin-binding protein family, C-terminal	0
PF01534	Frizzled/Smoothened family membrane region	6
PF01627	Hpt domain	0
PF02262	CBL proto-oncogene N-terminal domain 1	2
PF02377	Dishevelled specific domain	1
PF02761	CBL proto-oncogene N-terminus, EF hand-like	2
PF02762	CBL proto-oncogene N-terminus, SH2-like domain	2
PF02864	STAT protein, DNA binding domain	2
PF02865	STAT protein, protein interaction domain	2
PF03528	Rabaptin	0
PF03623	Focal adhesion targeting region	0
PF04692	Platelet-derived growth factor, N terminal	0
PF04709	Anti-Mullerian hormone, N terminal region	0
PF05196	PTN/MK heparin-binding protein family, N-terminal	0
PF05337	Macrophage colony stimulating factor-1	0
PF06554	Olfactory marker protein	0
PF07714	Tyrosine kinase	316
PF08916	Phenylalanine zipper	0
PF09034	TRADD, N-terminal domain	0
*	Interleukins	0

*PF00715, PF00727, PF01415, PF02025, PF03039 and PF07400

Figure 1

Click here to download Figure Figure_1.pdf

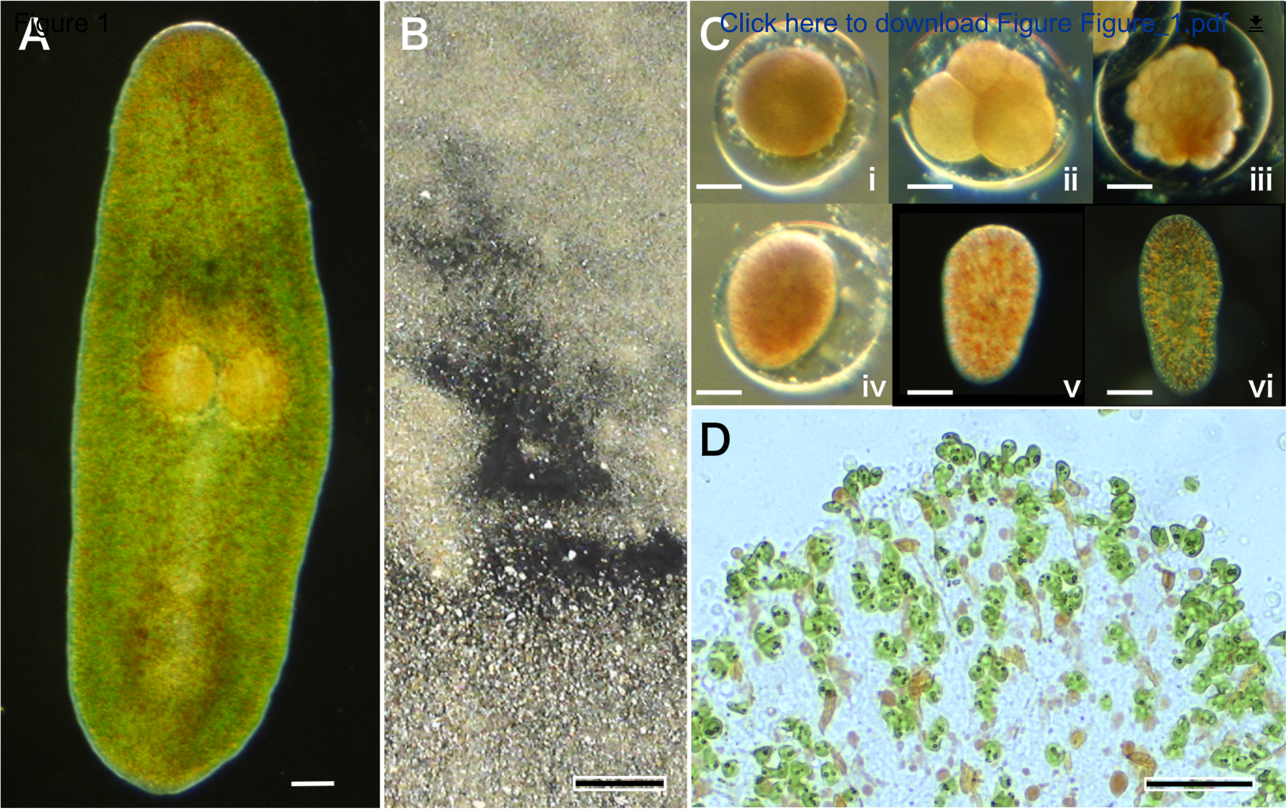

Supplementary Table 1

Click here to access/download
Supplementary Material
Table_S1.xlsx

Supplementary Table 2

Click here to access/download
Supplementary Material
Table_S2.xlsx

Supplementary Table 3

Click here to access/download
Supplementary Material
Table_S3.xlsx

Supplementary Figure 1

Click here to access/download
Supplementary Material
Figure_S1.pdf

Supplementary Figure 2

Click here to access/download
Supplementary Material
Figure_S2.pdf

Supplementary Figure 3

Click here to access/download
Supplementary Material
Figure_S3.pdf

Supplementary Figure 4

Click here to access/download
Supplementary Material
Figure_S4.pdf
