# Supplementary material for: A draft nuclear-genome assembly of the acoel flatworm Praesagittifera naikaiensis
Source: Gigascience. 2019 Apr 6;8(4):giz023. doi: 10.1093/gigascience/giz023 (PMC6451197; doi:10.1093/gigascience/giz023)
Supplement: GIGA-D-18-00363_Revision_1.pdf [file giz023_giga-d-18-00363_revision_1.pdf]

GigaScience

A draft genome assembly of the acoel flatworm Praesagittifera naikaiensis

--Manuscript Draft--

**Manuscript Number:**	GIGA-D-18-00363R1
**Full Title:**	A draft genome assembly of the acoel flatworm Praesagittifera naikaiensis
**Article Type:**	Data Note
**Funding Information:**	Research Institute of Marine Invertebrates (No fund number) — Dr. Akira Hikosaka<br>Japan Society for the Promotion of Science (17K07535) — Dr. Akira Hikosaka<br>Okinawa Institute of Science and Technology Graduate University (Internal Fund) — Dr. Noriyuki Satoh
**Abstract:**	Background<br><br>Acoels are primitive bilaterians with very simple soft-bodies, in which many organs, including the gut, are not developed. They provide platforms for studying molecular and developmental mechanisms involved in formation of the basic bilaterian body-plan, whole-body regeneration, and symbiosis with photosynthetic microalgae. Because genomic information is essential for future research on acoel biology, we sequenced and assembled the nuclear genome of an acoel, Praesagittifera naikaiensis.<br><br>Findings<br><br>To avoid sequence contamination derived from symbiotic microalgae, DNA was extracted from embryos that were free of algae. More than 290x sequencing coverage was achieved using a combination of Illumina (paired end and mate-pair libraries) and PacBio sequencing. RNA-seq and Iso-Seq data from embryos, larvae, and adults were also obtained. First, a preliminary ~17-kbp mitochondrial genome was assembled, which was deleted from the nuclear sequence assembly. As a result, a draft nuclear-genome assembly was ~656-Mbp in length, with a scaffold N50 of 117 kb and a contig N50 of 57 kb, respectively. Although ~70% of the assembled sequences were likely comprised of repetitive sequences that include DNA transposons and retrotransposons, the draft genome was estimated to contain 22,143 protein-coding genes, approximately 99% of which were substantiated by corresponding transcripts. We could not find horizontally-transferred microalgal genes in the acoel genome. BUSCO analyses indicated that 77% of the conserved single-copy genes were complete. Pfam domain analyses provided a basic set of gene families for transcription factors and signaling molecules.<br><br>Conclusions<br><br>Our present sequencing and assembly of the P. naikaiensis nuclear genome are comparable to those of other metazoan genomes, providing basic information for future studies of genic and genomic attributes of this animal group. Such studies may shed light on the origins and evolution of simple bilaterians.
**Corresponding Author:**	Asuka Arimoto<br><br>JAPAN
**Corresponding Author Secondary Information:**	
**Corresponding Author's Institution:**	
**Corresponding Author's Secondary Institution:**	
**First Author:**	Asuka Arimoto

Powered by Editorial Manager® and ProduXion Manager® from Aries Systems Corporation

**First Author Secondary Information:**	
**Order of Authors:**	Asuka Arimoto
	Tomoe Hikosaka-Katayama
	Akira Hikosaka
	Kunifumi Tagawa
	Toyoshige Inoue
	Tatsuya Ueki
	Masa-aki Yoshida
	Miyuki Kanda
	Eiichi Shoguchi
	Kanako Hisata
	Noriyuki Satoh
**Order of Authors Secondary Information:**	
**Response to Reviewers:**	Reviewer reports:

Reviewer #1: The manuscript "A draft genome assembly of the acoel flatworm Praesagittifera naikaiensis" presents the 654 Mbp assembly for this flatworm. The genome appears to be assembled well, with good depth and using both Illumina and Pacbio reads for assembly, as well as RNA-seq for annotation.

(1-1) BUSCO analyses supported completeness of 77% of the annotated genes. BUSCO can also be ran against the genome assembly. This may be why your CEGMA numbers were substantially higher. Also, as reported in a recent study (https://www.nature.com/articles/s41588-018-0262-1/) there are some 7 "core" CEGMA genes that are consistently missing across all trematodes, suggesting that the BUSCO completeness may be higher than estimated, since there are likely some "core" functions that are legitimately absent from Praesagittifera naikaiensis. It may also be provided some "core" functions from its symbiosis with micro algae.

We appreciate the reviewer’s comments. First, our BUSCO data were obtained by running BUSCO against the genome assembly. BUSCO analysis is carried out using metazoan genes while CEGMA against eukaryote genes. At present, we cannot explain the reason why the BUSCO score is lower than that of CEGMA, although they are similar. To avoid confusion between BUSCO and CEGMA results, we only used BUSCO analysis in the revised manuscript.
As to the comment that some core genes are consistently missing across all trematodes, our research group is now conducting a genome decoding project of a parasitic mesozoan, in which we found many lost genes in basic metabolic pathways. However, we failed to find such gene loss in this acoel genome.
Sorry but we cannot understand well the meaning of your comment, “It may also be provided some "core" functions from its symbiosis with micro algae”. Regarding this, we carefully examined a possible mixture of algal genes in the acoel genome assembly. First, to avoid contamination of algal DNA, we used, as mentioned in “Biological materials”, embryonic cells which do not contain symbiotic algae. Therefore, basically our data came from the acoel itself. Second, as you might mentioned, there is a possibility of horizontal transfer of microalgal genes into the acoel genome. In order to check whether the assembled genome contains sequences of photosynthetic organisms, we carried out blastx analysis of the assembled genome against NCBI NR database to find sequences with similarity to those of photosynthetic organisms. However, no such data were obtained. This convinces us that our draft assembly does not contain algal genes, although we have no idea at present on a possibility that some acoel core-functions depend on symbiotic algae.

(1-2) For tables 3 and 4, you could exclude all the entries with zero count.

Powered by Editorial Manager® and ProduXion Manager® from Aries Systems Corporation

Accordingly, we excluded the entries with zero counts from Tables 3 and 4.

(1-3) The genomes of S. roscoffensis and the xenoturbellid X. bocki are available. For the sake of evaluation and comparison of this genome, it would be very good to have a table comparing the basic statistics of these species (and any other xenacoelomorph species available), such as total length, protein coding genes, completeness, N50, etc. This would help to place this genome in the context of other available genomes and would help readers better connect resources in the future.

Probably due to the brevity of the description in the original version, we suspect that the reviewer misunderstood the present status of research in this field. That is, the present study is the first acoel "nuclear" genome, but not the first "mitochondrial" genome. Yes, there are reports of mitochondrial genomes of several acoel species, including S. roscoffensis, and X. bocki as well, but no nuclear genomes. Therefore, we cannot provide a table for genome comparison as the reviewer suggested. However, again, this is partially because our previous description was inadequate. We have revised the manuscript to distinguish clearly between nuclear and mitochondrial genomes (pages 2, 3, 5, and 7).

Reviewer #2: The authors collected genomic and transcriptomic data for the acoelomate worm Praesagittifera naikaiensis. The species belongs to an important group of organisms that are key to understanding the origin of bilateral body plan, the ability of whole-body regeneration, and symbiosis with photosynthetic microalgae. Genomic resources for this an organism will help these key areas of research.

The authors used Pacific Biosciences long reads and Illumina paired end short reads for both genomic and transcriptomic data sets. They used a hybrid approach for de novo assembly and Iso-seq for validation of the transcripts predicted with the RNAseq data. I have some minor concerns and suggestions regarding the assembly approach and presentation of the paper:

(2-1) The authors collected high coverage (73X) PacBio reads for genome assembly. At this coverage, a PacBio only assembler is likely to produce a more contiguous and accurate assembly (e.g. see https://academic.oup.com/nar/article/44/19/e147/2468393). Given that a heterozygous sample was sequenced, Falcon could be used as the PacBio only assembler. I was also wondering if the authors tried the hybrid assembler DBG2OLC (and Platanus as the Illumina assembler as described in https://academic.oup.com/nar/article/44/19/e147/2468393) which often works better than Masurca?

We appreciate the reviewer's comments on the methodology of genome assembly. Our research group has so far sequenced genomes of more than 10 animal taxa. The assembly is affected by the choice of Illumina and/or PacBio platform, or their combination; therefore, we examined various methods including those the reviewer suggested. For example, we tried the FALCON assembler using subreads with more than 2 kb of PacBio, but the total assembled length resulted in only 2.6 Mb. We have obtained 73X PacBio data, but those more than 5 kb were only 20X. Another cause might come from sampling of embryos from different batches (it is impossible to obtain enough samples from a single individual). We also attempted to use a hybrid assembler DBG2OLC with Platanus to obtain a better contig assembly. The most suitable parameter usage gave rise to a 630 Mb assembly with scaffold number 11 million and scaffold N50 = 50 bp. Namely, compared to MaSuRCA, all these scaffolds were very fragmented.

(2-2) The authors used Racon to polish the assembly with long reads. However, Quiver or Arrow is recommended over Racon for polishing PacBio assemblies. With 70X coverage, Arrow (and Quiver) can achieve higher consensus accuracy than Racon.

As mentioned above, probably due to mixed embryonic samples from different batches, our PacBio reads did not always provide data useful for further analysis, such

as with Arrow. However, as mentioned above, we tried various polishing methods, and Racon combined with Pilon resulted in the best assembly; thus, we presented data resulting from this method.

(2-3) On line 195, "others" is mentioned as if it is a type of TE. It would be more appropriate to mention them as unclassified. On a related note, all repeats appear to consist of only TEs. Do these worms not have any simple or Low complexity repeats?

We appreciate this comment. Accordingly, we changed the description of “others” to more explicit language, including simple repeats. We also explained more clearly the rate and types of TEs. (Page 8, lines 205-215)

(2-4) The statements on the relationships between single copy and double copy genes and BUSCO and CEGMA were unclear (Line 201-204). The BUSCO and CEGMA both report the single copy and double copy genes based on their database and the percentages are based on the number of conserved genes they have searched from their database. It would be helpful to clarify these.

Good comment. Accordingly, we revised Table 1 (more simple form), in which “single and double copy genes” were deleted. In the revised form, we removed the CEGMA data in order to avoid confusion between BUSCO and CEGMA data. (Page 9, lines 231-232)

(2-5) One interesting analysis that the authors could do is to check the number of TEs that are located within the introns and the number of introns that are only TEs (intron length = TE length).

This was an interesting suggestion so that we checked it. We found that 32,110 TEs are present in intron regions; 29%, 18%, and 12% of them are correspond to “uncharacterized”, “LTR (Gypsy)” and DNA transposon (MULE), respectively. On the other hand, we failed to find introns that comprise only TEs. We have now included this result. (Page 8, lines 216-220).

(2-6) The authors mention that the adult worms carry symbiotic algae. I am curious to know whether the authors found any sequence reads that are derived from symbiotic algae. It would be nice to get this information. Similarly, does any of the contigs belong to symbiotic algae?

As the reviewer pointed out, the adult worms carry symbiotic algae. To avoid contamination from algal DNA, we used embryonic cells that do not contain symbiotic algae. Therefore, basically our data came from the acoel itself. However, as you mentioned, there is a possibility of horizontal transfer of microalgal genes into the acoel genome or contamination of algae during sampling procedure. In order to check whether the assembled genome contains sequences of photosynthetic organisms, we carried out blastx analysis of the assembled genome against NCBI NR database to find sequences with similarity to those of photosynthetic organisms. However, no such data were obtained. This convinces us that our draft assembly does not contain algal genes.

(2-7) I could not access the genome browser at the marinegenomics website the authors have provided. Is the link correct?

We apologize for the inconvenience. We will open the genome browser if our manuscript is accepted. However, an account for reviewing is available now. Reviewers can login to the browser at http://marinegenomics.oist.jp/gallery/users/sign_in with account ID: acoel-pna, and password: acoel-genome. We added in the revised form more clearly the genome browser information (Figure 2).

	(2-8) On Line 162, the sentence that starts with "parallel" looks incomplete and needs to be revised.<br>---------------<br>Sorry. We changed "Parallel" to "In addition," (line 165).
Additional Information:	
Question	Response
Are you submitting this manuscript to a special series or article collection?	No
**Experimental design and statistics**<br><br>Full details of the experimental design and statistical methods used should be given in the Methods section, as detailed in our Minimum Standards Reporting Checklist. Information essential to interpreting the data presented should be made available in the figure legends.<br><br>Have you included all the information requested in your manuscript?	Yes
**Resources**<br><br>A description of all resources used, including antibodies, cell lines, animals and software tools, with enough information to allow them to be uniquely identified, should be included in the Methods section. Authors are strongly encouraged to cite Research Resource Identifiers (RRIDs) for antibodies, model organisms and tools, where possible.<br><br>Have you included the information requested as detailed in our Minimum Standards Reporting Checklist?	Yes
**Availability of data and materials**<br><br>All datasets and code on which the conclusions of the paper rely must be either included in your submission or deposited in publicly available repositories (where available and ethically appropriate), referencing such data using	Yes

a unique identifier in the references and in the “Availability of Data and Materials” section of your manuscript.<br><br>Have you have met the above requirement as detailed in our Minimum Standards Reporting Checklist?	

Manuscript

Click here to view linked References

Click here to access/download;Manuscript;MainText_Revised.190110-

A draft nuclear-genome assembly of the acoel flatworm *Praesagittifera naikaiensis*

Asuka Arimoto[1+*], Tomoe Hikosaka-Katayama[2+], Akira Hikosaka[3], Kunifumi Tagawa[4], Toyoshige Inoue[4], Tatsuya Ueki[4,5], Masa-aki Yoshida[6], Miyuki Kanda[7], Eiichi Shoguchi[1], Kanako Hisata[1] and Noriyuki Satoh[1*]

[1]Marine Genomics Unit, Okinawa Institute of Science and Technology Graduate University, Onna, Okinawa 904-0495, Japan

[2]Natural Science Center for Basic Research and Development Center for Gene Science Division, Hiroshima University, Higashi-Hiroshima, Hiroshima 739-8527, Japan

[3]Division of Human Sciences, Graduate School of Integrated Arts and Sciences, Hiroshima University, Higashi-Hiroshima, Hiroshima 739-8521, Japan

[4]Marine Biological Laboratory, Graduate School of Science, Hiroshima University, Onomichi, Hiroshima 722-0073, Japan

[5]Department of Biological Science, Graduate School of Science, Hiroshima University, Higashi Hiroshima, Hiroshima 739-8526, Japan

[6]Marine Biological Science Section, Education and Research Center for Biological Resources, Faculty of Life and Environmental Science, Shimane University, Kamo 194, Okinoshima-cho, Oki, Shimane, 685-0024, Japan

[7]DNA Sequence Section, Okinawa Institute of Science and Technology Graduate University, Onna, Okinawa 904-0495, Japan

[+] These authors contributed equally

*Corresponding authors: Asuka Arimoto (asuka.arimoto@oist.jp), Noriyuki Satoh (norisky@oist.jp)

ORCID IDs:

Asuka Arimoto: 0000-0001-8220-5920; Tatsuya Ueki: 0000-0002-3112-9507; Masa-aki Yoshida: 0000-0002-5221-0320; Miyuki Kanda: 0000-0001-6410-9441; Eiichi Shoguchi: 0000-0003-3136-5558; Kanako Hisata: 0000-0002-6313-3340; Noriyuki Satoh: 0000-0003-4560-9250

Abstract

Background: Acoels are primitive bilaterians with very simple soft-bodies, in which many organs, including the gut, are not developed. They provide platforms for studying molecular and developmental mechanisms involved in formation of the basic bilaterian body-plan, whole-body regeneration, and symbiosis with photosynthetic microalgae. Because genomic information is essential for future research on acoel biology, we sequenced and assembled the nuclear genome of an acoel, *Praesagittifera naikaiensis*.

Findings: To avoid sequence contamination derived from symbiotic microalgae, DNA was extracted from embryos that were free of algae. More than 290x sequencing coverage was achieved using a combination of Illumina (paired end and mate-pair libraries) and PacBio sequencing. RNA-seq and Iso-Seq data from embryos, larvae, and adults were also obtained. First, a preliminary ~17-kbp mitochondrial genome was assembled, which was deleted from the nuclear sequence assembly. As a result, a draft nuclear-genome assembly was ~656-Mbp in length, with a scaffold N50 of 117 kb and a contig N50 of 57 kb, respectively. Although ~70% of the assembled sequences were likely comprised of repetitive sequences that include DNA transposons and retrotransposons, the draft genome was estimated to contain 22,143 protein-coding genes, approximately 99% of which were substantiated by corresponding transcripts. We could not find horizontally-transferred microalgal genes in the acoel genome. BUSCO analyses indicated that 77% of the conserved single-copy genes were complete. Pfam domain analyses provided a basic set of gene families for transcription factors and signaling molecules.

Conclusions: Our present sequencing and assembly of the *P. naikaiensis* nuclear genome are comparable to those of other metazoan genomes, providing basic information for future studies of genic and genomic attributes of this animal group. Such studies may shed light on the origins and evolution of simple bilaterians.

Key words: Acoel, *Praesagittifera naikaiensis,* Draft nuclear genome, ~22,000 predicted genes

Data description

Background

Acoels are small, very simple, planula-like animals lacking a coelom, a gut, and a central nervous system. Traditional taxonomy positioned the Acoela as the most basal order of the phylum Platyhelminthes [1]. Recent analyses using molecular data, however, have suggested that acoels are members of a new phylum, the Xenacoelomorpha, together with nemertodermatids and xenoturbellids [2, 3, 4]. However, whether Xenacoelomorpha is a monophyletic taxon, whether xenacoelomorphs are basal to all other bilaterians, and whether they have close affinity to ambulacrarians are matters of debate [2, 3, 4]. Nonetheless, acoels are pivotal to understanding the origins and evolution of bilaterians. Acoels also provide a platform for molecular studies of whole-body regeneration [5] and symbiosis with photosynthetic microalgae. Although mitochondrial genomes of four acoel species have been reported [6, 7, 8], their nuclear genomes have not been explored yet. Because nuclear genome information is essential to investigate biological questions regarding acoels, we sequenced and assembled a draft nuclear genome of the acoel, *Praesagittifera naikaiensis* (urn:lsid:marinespecies.org:taxname:379972).

Sampling and sequencing

Biological materials

The marine acoel worm, *Praesagittifera naikaiensis,* is 2-3 mm in length (Fig. 1A) [9]. Members of this species are easily found at seashores of the Seto Inland Sea, especially during the early summer season (Fig. 1B). Adults contain symbiotic microalgae, *Tetraselmis* sp., which are integrated during juvenile growth (Fig. 1C). Adults were collected at the seashore near the Marine Biological Laboratory of Hiroshima University and maintained in aquaria in the laboratory on a 12-h light/12-h dark photoperiod. Naturally laid eggs were collected and cultured for embryogenesis (Fig. 1C). Embryos were free of symbiotic microalgae. After washing embryos with filtered seawater, genomic DNA was extracted from them using the phenol/chloroform extraction.

Embryos, juveniles and adults were sampled for RNA sequencing. Total RNA extraction was performed using TRIzol Reagent (Invitrogen, 15596-026) and an RNeasy mini Kit (Qiagen, 74104).

Library preparation and sequencing

DNA: All sequencing libraries were constructed according to the manufacturers' standard protocols. Briefly, for the Illumina platform, PCR-free, paired-end libraries were prepared using an Illumina TruSeq DNA PCR-Free LT Library Prep Kit (Illumina, FC-121-3001). Four mate-pair libraries were prepared using a Nextera Mate Pair Library Prep Kit (Illumina, FC-132-1001) (Additional file 1).

For the PacBio platform, a DNA library was prepared using the manufacturer's 10-kb template preparation protocol. A SMRTbell Template Prep Kit 1.0 (Pacific Biosciences, 100-259-100) was used for PacBio library preparation. The long-read DNA library was sequenced using a PacBio RSII sequencer employing P6-C4 chemistry (Pacific Biosciences, 100-372-700) with 360 min movie lengths. A total of 52 SMRT Cells were sequenced for long-read DNA library.

RNA: An RNA-seq library was prepared using a TruSeq Stranded mRNA LT Sample Prep Kit (Illumina, RS-122-2101). The library was sequenced using the Illumina HiSeq 2500 platform (Additional file 1).

cDNAs for Iso-Seq libraries were prepared using a SMARTer PCR cDNA synthesis kit (Clontech, 634925). The SageELF size selection system (Sage Science, MA, USA) was used following the manufacturer's standard protocol (Additional file 1). A SMRTbell Template Prep Kit 1.0 (Pacific Biosciences, 100-259-100) was used for Iso-Seq library preparation. The library was sequenced using a PacBio RSII sequencer employing P6-C4 chemistry (Pacific Biosciences, 100-372-700) with 360 min movie lengths. A total of 8 SMRT Cells were sequenced for the Iso-Seq RNA library.

Assembly of mitochondrial and nuclear genomes

Adapter sequences in PCR-free and mate-pair Illumina reads were removed with Trimmomatic 0.36 (Trimmomatic, RRID:SCR_011848) [10] and NextClip 1.3.1 (NextClip, RRID:SCR_005465) [11], respectively. Low-quality (<Q20) inserts were removed using Sickle 1.33 (Sickle, RRID:SCR_006800) [12] after adapter cleanup. Reads that lacked a corresponding pair were discarded.

Mitochondrial genome assembly: To distinguish mitochondrial genome sequences from the nuclear genome assembly, we first assembled the mitochondrial genome. To this end, a mitochondrial 16S ribosomal RNA of *Symsagittifera roscoffensis* (accession number NC_014578) [7] was used to collect PacBio long-reads of *P. naikaiensis* sequences using BLAST+ 2.3.0 [13] with the "dc-megablast" option.

Collected reads longer than 1 kb and shorter than 12 kb were assembled using sprai 0.9.9.19 (http://zombie.cb.k.u-tokyo.ac.jp/sprai/index.html) with default settings. Circularity of assembled contigs was checked automatically in the sprai assembly pipeline.

Nuclear genome assembly: *P. naikaiensis* mitochondrial sequences were mapped onto trimmed reads using BWA 0.7.12 (BWA, RRID:SCR_010910) [14] and those read

pairs that mapped onto the mitochondrial sequences were excluded from the dataset. PacBio long-reads were also mapped against the mitochondrial genome using BLASR (BLASR, RRID:SCR_000764) (commit version: 5.3.574e1c2) [15]. Only unmapped or cleaned-up Illumina and PacBio reads were assembled using the MaSuRCA (MaSuRCA, RRID:SCR_010691) assembler 3.2.2 [16].

Putative heterozygous and/or polymorphic sequences that remained in the assembled genome were merged as homozygous sequences using redundans 0.13c [17]. Gaps in the homozygous genome were filled using PBJelly (PBJelly, RRID:SCR_012091) in PBSuite 15.8.24 [18]. After gap closing, BESST 2.2.6 [19] and LINKS 1.8.5 [20] were used to perform scaffolding with Illumina and PacBio reads, respectively. Scaffolds were polished using Racon (commit version: 083444) [21] with PacBio long reads.

PacBio Iso-Seq reads were mapped onto scaffolds using GMAP (GMAP, RRID:SCR_008992) version 2017-08-15 [22], and then L_RNA_scaffolder [23] was used to concatenate scaffolds based on the results of Iso-Seq read mapping. Scaffolds were polished using Pilon 1.22 (Pilon, RRID:SCR_014731) [24] with PCR-free Illumina reads used for MaSuRCA assembly. BUSCO 3.0.2 (BUSCO, RRID:SCR_015008) [25] with a metazoan dataset was used to evaluate the polished final genome assembly.

Genome size estimation

PCR-free, paired-end reads used for genome assembly were analyzed. K-mers in the dataset were counted with Jellyfish 2.2.3 (Jellyfish, RRID:SCR_005491) [26] (Additional file 2). The genome size of *P. naikaiensis* was estimated from obtained k-mer frequencies using GenomeScope web tools [27].

Repeat analysis

Repetitive sequences in the assembled genome were identified using RepeatModeler 1.0.11 (RepeatModeler, RRID:SCR_015027) [28] and RepeatMasker 4.0.7 (RepeatMasker, RRID:SCR_012954) [29].

Transcriptome assembly, gene prediction, and gene annotation

Adapter sequences and low-quality (<Q30) reads in the resulting RNA-seq paired-end data were removed using Trimmomatic (Trimmomatic, RRID:SCR_011848). Cleaned reads were assembled using Trinity 2.1.1 (Trinity, RRID:SCR_013048) [30] with default settings and the strand-specific option. In addition, genome-guided transcriptome assembly was performed. RNA-seq reads were mapped onto the genome using STAR 2.5.2a (STAR, RRID:SCR_015899) [31] and then mapped reads were assembled using Trinity. *De novo* assembled Illumina transcriptome and PacBio Iso-Seq sequences were mapped onto the genome using minimap-2 version 2.6 [32] with the "-ax splice" option. These mapping results and the genome-guided assembly of Illumina RNA-seq reads were integrated based on genome sequences using PASA 2.2.0 (PASA, RRID:SCR_014656) [33]. Putative full-length (FL) transcripts having both a 5' UTR and a 3' UTR were detected using TransDecoder 5.0.2 [34]. These FL transcripts were used as a training set for gene prediction. *De novo* transcriptome assembly of a dataset containing 15 xenacoelomorphs (Additional file 3) was also performed, following the procedure described above to create similarity hints for gene prediction. Assembled sequences of other acoels were translated into protein sequences using TransDecoder and then mapped against the *P. naikaiensis* genome using Exonerate 2.2.0 (Exonerate, RRID:SCR_016088) [35]. A final set of gene models reflecting hint information was generated with AUGUSTUS 3.2.1 (Augustus, RRID:SCR_008417) [36]. Gene models were annotated using BLAST searches (E-value cutoff of 10^{-5}) against the NCBI RefSeq protein database release 88. Protein domains in gene models were detected using HMMER 3.1b2 (Hmmer, RRID:SCR_005305) [37] and Pfam-A 31.0 under default settings, except for an E-value cutoff of 10^{-5}.

A draft assembly

Mitochondrial genome: The complete, closed circular mitochondrial genome of *P. naikaiensis* was recovered from genome sequencing data. The mitochondrial genome is 17,787 nucleotides long, and contains 12 protein-coding genes, small and large rRNAs and 22 predicted tRNAs (Additional file 4). When *cox1* was positioned at the start of the genome on the 'positive' strand, eight protein-coding genes were found in the same strand while *nad2*, *cytb* and *nad5* were found on the 'negative' strand (Additional file 4). Both rRNAs were found separately on the positive strand. Although the number of mitochondrial genes of *P. naikaiensis* is comparable to that of the previously reported mitochondrial genes of *Archaphanostoma ylvae* [8], the order of genes in the genomes was quite different between them.

Nuclear genome: K-mer analysis showed that the *P. naikaiensis* genome constitutes approximately 654 Mb (Table 1; Additional file 2). Illumina paired-end and mate-pair, and PacBio reads provided 204x and 221x, and 73x coverage of the estimated genome, respectively (Additional files 1 and 2). The assembly appeared to plateau during both scaffolding and contig formation (Additional file 5). As a result, the draft assembly comprised 656 Mb (Table 1), very close to the estimated genome size. The scaffold N50 reached 117 kb, and 12 scaffolds were over 500 kb in length (Table 1; Additional file 6). Inserted gaps composed only 1.7% of the total scaffold assembly (Additional file 6). The contig N50 was 57 kb, and 41 contigs exceeded 250 kb (Table 1; Additional file 6). The GC content of the genome was estimated 39.1% (Table 1; Additional file 7).

Analysis of repetitive sequences showed that approximately 69.8% of the genome consists of repetitive sequences (Table 1 and Table 2, this value was estimated by deletion of overlapped sequences from the total data). DNA transposons (MULE, Marverick, hAT and others), retrotransposons (LTR, LINE and SINE), and other repetitive sequences (including low complexity repeats and simple repeats) represented 12.2%, 41.5%, and 2.2% of the assembly, respectively (Table 2). The most prominent

family was Gypsy of LTR (long terminal repeat), occupying 28.2% of the genome. In addition, the genome contains unclassified repetitive elements that accounted for 20.4% of it (Table 2). In addition, unclassified repeats occupied 20.4% of the genome. Thus, the *P. naikaiensis* genome contains a comparatively high percentage of repetitive sequences.

An interesting question concerns the locations of transposable elements (TEs) in introns. We found that 32,110 TEs are present in intron regions; 29%, 28%, and 12% of them correspond to "uncharacterized," "LTR (Gypsy)," and "DNA transposon (MULE)," respectively. On the other hand, we failed to find introns that are composed of only TEs.

Transcriptomes

Transcriptome data, especially those from PacBio Iso-Seq long-reads, provided a set of high-quality RNA data (Additional file 1). An average length of transcriptomes was 2,447 nucleotides, and an average number of exons per gene was 5.7 (Table 1).

Gene modeling

Gene modeling of the *P. naikaiensis* genome produced 22,143 protein-coding genes (Table 1). As mentioned above, we obtained a set of high-quality RNA data. As a result, 99% of gene models were substantiated by the transcriptomes (Table 1).

BUSCO analysis indicated that 76.5% and 3.8% of them were supported as complete and fragmented genes, respectively (Table 1).

Gene annotation

Gene families predicted by RefSeq (BLAST), Pfam (HMMER) and PANTHER (HMMER) were 15,294, 13,225 and 17,384 in number, respectively (Additional file 8). Using Pfam-supported families, we examined the number of gene families. Table 3 shows numbers of putative transcription regulator genes in the *P. naikaiensis* genome.

The two most abundant families were Zinc-finger (C2H2 type) and homeobox domain-containing genes, with 73 and 62 members, respectively. Twenty each were annotated to the HLH and Zinc-finger (C4 types) families. Although more detailed analysis is required, the *P. naikaiensis* genome appears to contain numbers of transcription regulator genes comparable to those of other bilaterian genomes.

A similar analysis was carried out on putative signaling molecule genes (Table 4). The largest gene family was tyrosine kinase, represented by 316 genes. In addition, EGF-like domain genes, G-protein alpha subunit genes, and Regulator of G-protein signaling genes numbered 28, 31, and 16, respectively (Table 4).

Genome browser

A genome browser was established for the assembled sequences using the JBrowser 1.12.3 [38]. Its URL is http://marinegenomics.oist.jp/p_naikaiensis/viewer?project_id=71 (Fig. 2). The gene annotations from the protein domain search and BLAST search have similarly been shown on the site.

Availability of data

Genomic and transcriptomic sequence reads have been deposited in the DDBJ sequence read archive under accession number PRJDB7329. All data are also available from the *GigaScience* GigaDB repository [39].

Additional files

Additional file 1: Supplementary Table 1: Sequence data summary.

Additional file 2: Supplementary Figure 1: K-mer analysis and genome size estimation of *Praesagittifera naikaiensis* genomic DNA reads.

Additional file 3: Supplementary Table 2: Xenacoelomorph dataset used for gene prediction.

Additional file 4: Supplementary Figure 2: A preliminary circular assembly of the mitochondria genome of *Praesagittifera naikaiensis*.

Additional file 5: Supplementary Figure 3: Accumulation of assembled sequences (contigs, blue and scaffolds, red) reaching over 600 Mbp.

Additional file 6: Supplementary Table 3: Summary of the *Praesagittifera naikaiensis* genome assembly.

Additional file 7: Supplementary Figure 4: GC content of the *Praesagittifera naikaiensis* genome.

Additional file 8: Supplementary Figure 5: *Praesagittifera naikaiensis* gene annotation.

Competing interests

The authors declare that they have no competing interests.

Authors' contributions

NS, THK, AH, KT, AA, MAY, and TU conceived and supervised the project. THK and TI collected the majority of samples. MK and AA performed sequencing. AA, KH, ES and THK performed analyses. NS, AA, and THK prepared the manuscript and all authors approved the final manuscript.

Abbreviations

BUSCO： Benchmarking Universal Single-Copy Orthologs； FL： full-length； Gbp: Gigabase-pair; kbp: kilobase-pair; LTR: long terminal repeat； Mbp: megabase-pair; TE： transposable elements; UTR: Un-Translated Region

Acknowledgments

The work was funded by OIST Internal Funds to the Marine Genomics Unit (NS). This work was also supported by a JSPS grant (No.17K07535) and a research grant from the

Research Institute of Marine Invertebrates to AH. We are grateful to Dr. Steven D. Aird for his technical editing of the manuscript.

References

1. Hyman LH. The Invertebrates: Platyhelminthes and Rhynchocoela; the Acoelomate Bilateria. New York: McGraw-Hill; 1951.
2. Ruiz-Trillo I, Riutort M, Littlewood DTJ, Hejnol EA, Baguñà J. Acoel flatworms: earliest extant bilaterian metazoans, not members of Platyhelminthes. *Science* 1999;283:1919-23.
3. Philippe H, Brinkmann H, Copley RR, Moroz LL, Nakano H, Poustka AJ, et al. Acoelomorph flatworms are deuterostomes related to *Xenoturbella*. *Nature* 2011;470:255-8.
4. Cannon JT, Vellutini BC, Smith J 3rd, Ronquist F, Jondelius U, Hejnol A. Xenacoelomorpha is the sister group to Nephrozoa. *Nature* 2016;530:89-93.
5. Srivastava M, Mazza-Curll KL, van Wolfswinkel JC, Reddien PW. Whole-body acoel regeneration is controlled by Wnt and Bmp-Admp signaling. *Curr Biol*. 2014;24:1107-13.
6. Ruiz-Trillo I, Riutort M, Fourcade HM, Baguñà J, Boore JL.Mitochondrial genome data support the basal position of Acoelomorpha and the polyphyly of the Platyhelminthes. *Mol Phylogenet Evol*. 2004;33:321-32.
7. Mwinyi A, Bailly X, Boulat SJ, Jondelius, U, Littlewood DTJ, Podsiadlowski L. The phylogenetic position of Acoela as revealed by the complete mitochondrial genome of *Symsagittifera roscoffensis*. *BMC Biol*. 2010;10:309
8. Robertson HE, Lapraz F, Egger B, Telford MJ, Schiffer PH. The mitochondrial genomes of the acoelomorph worms *Paratomella rubra, Isodiametra pulchra and Archaphanostoma ylvae*. *Sci Rep*. 2017;12:1847.
9. Hikosaka-Katayama T, Hikosaka A. Artificial Rearing System for *Praesagittifera naikaiensis* (Acoela, Acoelomorpha). *Studies in Human Science* 2015;10:17-23.
10. Bolger AM, Lohse M, Usadel B. Trimmomatic: a flexible trimmer for Illumina sequence data. *Bioinformatics* 2014;30:2114-20.

11. Leggett RM, Clavijo BJ, Clissold L, Clark MD, Caccamo M. NextClip: an analysis and read preparation tool for Nextera Long Mate Pair libraries. *Bioinformatics* 2014; 30:566-8.
12. Sickle https://github.com/najoshi/sickle
13. Camacho C, Coulouris G, Avagyan V, Ma N, Papadopoulos J, Bealer K, et al. BLAST+: architecture and applications. *BMC Bioinformatics* 2009;10:421.
14. Li H. Aligning sequence reads, clone sequences and assembly contigs with BWA-MEM. 2013. arXiv:1303.3997v2 [q-bio.GN].
15. Chaisson MJ. Tesler G. Mapping single molecule sequencing reads using basic local alignment with successive refinement (BLASR): application and theory. *BMC Bioinformatics* 2012;13:238.
16. Zimin AV, Marçais G, Puiu D, Roberts M, Salzberg SL, Yorke JA. The MaSuRCA genome assembler. *Bioinformatics* 2013;29:2669-77.
17. Pryszcz LP, Gabaldón T. Redundans: an assembly pipeline for highly heterozygous genomes. *Nucleic Acids Res*. 2016;44:e113.
18. English AC, Richards S, Han Y, Wang M, Vee V, Qu J, et al. Mind the gap: upgrading genomes with Pacific Biosciences RS long-read sequencing technology. *PLoS One* 2012;7:e47768.
19. Sahlin K, Chikhi R, Arvestad L. Assembly scaffolding with PE-contaminated mate-pair libraries. *Bioinformatics* 2016;32:1925-32.
20. Warren RL, Yang C, Vandervalk BP, Behsaz B, Lagman A, Jones SJ, et al. LINKS: Scalable, alignment-free scaffolding of draft genomes with long reads. *Gigascience* 2015;4:35.
21. Vaser R, Sović I, Nagarajan N, Šikić M. Fast and accurate de novo genome assembly from long uncorrected reads. *Genome Res*. 2017;27:737-46.
22. Wu TD, Watanabe CK. GMAP: a genomic mapping and alignment program for mRNA and EST sequences. *Bioinformatics* 2005;21:1859-75.

23. Xue W, Li JT, Zhu YP, Hou GY, Kong XF, Kuang YY, et al. L_RNA_scaffolder: scaffolding genomes with transcripts. *BMC Genomics*. 2013;14:604.
24. Walker BJ, Abeel T, Shea T, Priest M, Abouelliel A, Sakthikumar S, et al. Pilon: an integrated tool for comprehensive microbial variant detection and genome assembly improvement. *PLoS One* 2014;9:e112963.
25. Waterhouse RM, Seppey M, Simão FA, Manni M, Ioannidis P, Klioutchnikov G, et al. BUSCO applications from quality assessments to gene prediction and phylogenomics. *Mol Biol Evol*. 2018;35:543-8.
26. Marçais G, Kingsford C. A fast, lock-free approach for efficient parallel counting of occurrences of k-mers. *Bioinformatics* 2011;27:764-70.
27. Vurture GW, Sedlazeck FJ, Nattestad M, Underwood CJ, Fang H, Gurtowski J, et al. GenomeScope: fast reference-free genome profiling from short reads. *Bioinformatics* 2017;33:2202-4.
28. RepeatModeler http://www.repeatmasker.org/RepeatModeler
29. RepeatMasker http://www.repeatmasker.org
30. Grabherr MG, Haas BJ, Yassour M, Levin JZ, Thompson DA, Amit I, et al. Full-length transcriptome assembly from RNA-Seq data without a reference genome. *Nat Biotechnol*. 2011;29:644-52.
31. Dobin A, Davis CA, Schlesinger F, Drenkow J, Zaleski C, Jha S, et al. STAR: ultrafast universal RNA-seq aligner. *Bioinformatics* 2013;29:15-21.
32. Li H. Minimap2: pairwise alignment for nucleotide sequences. *Bioinformatics* 2018. doi: 10.1093/bioinformatics/bty191.
33. Haas BJ, Delcher AL, Mount SM, Wortman JR, Smith RK Jr, Hannick LI, et al. Improving the *Arabidopsis* genome annotation using maximal transcript alignment assemblies. *Nucleic Acids Res*. 2003;31:5654-66.
34. TransDecoder https://github.com/TransDecoder/TransDecoder
35. Slater GS, Birney E. Automated generation of heuristics for biological sequence comparison. *BMC Bioinformatics* 2005;6:31.

36. Stanke M, Diekhans M, Baertsch R, Haussler D. Using native and syntenically mapped cDNA alignments to improve de novo gene finding. *Bioinformatics* 2008;24:637-44.
37. HMMER http://hmmer.org
38. Skinner ME, Uzilov AV, Stein LD, Mungall CJ, Holmes IH. JBrowse: a next-generation genome browser. *Genome Res*. 2009;19:1630-1638.
39. Arimoto A; Hikosaka-Katayama T; Hikosaka A; Tagawa K; Inoue T; Ueki T; Yoshida M; Kanda M; Shoguchi E; Hisata K; Satoh N (2019): A draft genome assembly of the acoel flatworm Praesagittifera naikaiensis. *GigaScience* Database. http://dx.doi.org/10.5524/100564

Figure Legends

Figure 1: The acoel worm, *Praesagittifera naikaiensis.* (A) An adult, dorsal view. Anterior, top and posterior, bottom. Green dots throughout the entire body are symbiotic green algae. Two eggs are seen in the center of the worm. (B) An enormous number of adults gathering on the sandy seashore, resembling dark masses. (C) Embryogenesis. A newly laid egg within the eggshell (i), a four-cell stage embryo (ii), a gastrula (iii), a flattened-stage embryo (iv), newly-hatched aposymbiotic algae (v), and a symbiotic juvenile with symbiotic algae (vi). (D) A peripheral region of an adult worm showing symbiotic microalgae, *Tetraselmis* sp. Scale = 50 μm in (A), (C), (D); 10 cm in (B).

Figure 2: A shot of the genome browser of *Praesagittifera naikaiensis.* Its URL is http://marinegenomics.oist.jp/p_naikaiensis/viewer?project_id=71.

Table 1: Genome assembly statistics

Genome features	*Praesagittifera naikaiensis*
Estimated genome size*	654.1 Mb
Assembled genome size	656.1 Mb
Scaffolds (≥500 bp)	
Number	12,072
N50	117 kb
Contigs (≥500 bp)	
Number	24,071
N50	57 kb
Gaps	1.66%
Repetitive sequences	69.8%
GC content	39.1%
Predicted protein-coding genes (loci)	22,143
Genes with transcript support	99%
Mean transcript length	2,447 nt
Mean exon frequency per gene	5.7
BUSCO analysis	
Complete	748/978 (76.5%)
Fragmented (only)	37/978 (3.8%)

* Estimated by k-mer analysis of Illumina PCR-free reads as shown Supplementary Figure 1.

Table 2: Repetitive sequences in the *Praesagittifera naikaiensis* genome

Class			Percentage in the assembly
DNA transposons			12.2
		MULE	5.7
		Maverick	4.5
		hAT	0.9
		Others	1.1
Retrotransposons			41.5
	LTR		35.7
		Gypsy	28.2
		Copia	1.5
		Pao	1.1
		Others	4.9
	LINE		4.8
		CR1	1.7
		CRE	1.0
		L2	0.9
		Others	1.2
	SINE		1.0
Others			2.2
	RNA		0.03
	Low complexity		0.07
	Satellite		0.4
	Simple repeat		1.7
Unclassified			20.4
Total (overlapped sequences were excluded from a total of repetitive sequences)			69.8

Table 3: Numbers of putative transcriptional regulator genes in the *Praesagittifera naikaiensis* genome

Accession	ID	Description	No. of genes
PF00010	HLH	Helix-loop-helix DNA-binding domain	20
PF00046	Homeobox	Homeobox domain	62
PF00096	zf-C2H2	Zinc finger, C2H2 type	73
PF00104	Hormone_recep	Ligand-binding domain of nuclear hormone	14
PF00105	zf-C4	Zinc finger, C4 type	20
PF00157	Pou	Pou domain	3
PF00170	bZIP_1	bZIP transcription factor	13
PF00178	Ets	Ets-domain	13
PF00250	Fork_head	Fork head domain	11
PF00292	PAX	'Paired box' domain	5
PF00319	SRF-TF	SRF-type transcription factor	2
PF00320	GATA	GATA zinc finger	7
PF00505	HMG_box	HMG (high mobility group) box	12
PF00554	RHD	Rel homology domain (RHD)	1
PF00853	Runt	Runt domain	1
PF00870	P53	P53 DNA-binding domain	1
PF00907	T-box	T-box	4
PF01388	ARID	ARID/BRIGHT DNA-binding domain	4
PF01530	zf-C2HC	Zinc finger, C2HC type	2
PF02376	CUT	CUT domain	3
PF03299	TF_AP-2	Transcription factor AP-2	1
PF05044	Prox1	Homeo-prospero domain	1
PF07527	Hairy_orange	Hairy Orange	1
PF07716	bZIP_2	Basic region leucine zipper	11

Table 4: Numbers of genes encoding putative signaling molecules in the *Praesagittifera naikaiensis* genome

Accession	ID	Description	No. of genes
PF00008	EGF	EGF-like domain	28
PF00019	TGF_beta	Transforming growth factor beta like	5
PF00110	wnt	wnt family	4
PF00167	FGF	Fibroblast growth factor	3
PF00219	IGFBP	Insulin-like growth factor binding protein	1
PF00503	G-alpha	G-protein alpha subunit	31
PF00615	RGS	Regulator of G protein signaling	16
PF00631	G-gamma	GGL domain	5
PF00688	TGFb_propeptide	TGF-beta propeptide	3
PF00778	DIX	DIX domain	5
PF01017	STAT_alpha	STAT protein, all-alpha domain	2
PF01534	Frizzled	Frizzled/Smoothened family membrane region	6
PF02262	Cbl_N	CBL proto-oncogene N-terminal domain 1	2
PF02377	Dishevelled	Dishevelled specific domain	1
PF02761	Cbl_N2	CBL proto-oncogene N-terminus, EF hand-like	2
PF02762	Cbl_N3	CBL proto-oncogene N-terminus, SH2-like domain	2
PF02864	STAT_bind	STAT protein, DNA binding domain	2
PF02865	STAT_int	STAT protein, protein interaction domain	2
PF07714	Pkinase_Tyr	Tyrosine kinase	316

Figure 1

Click here to access/download;Figure;Figure_1.pdf

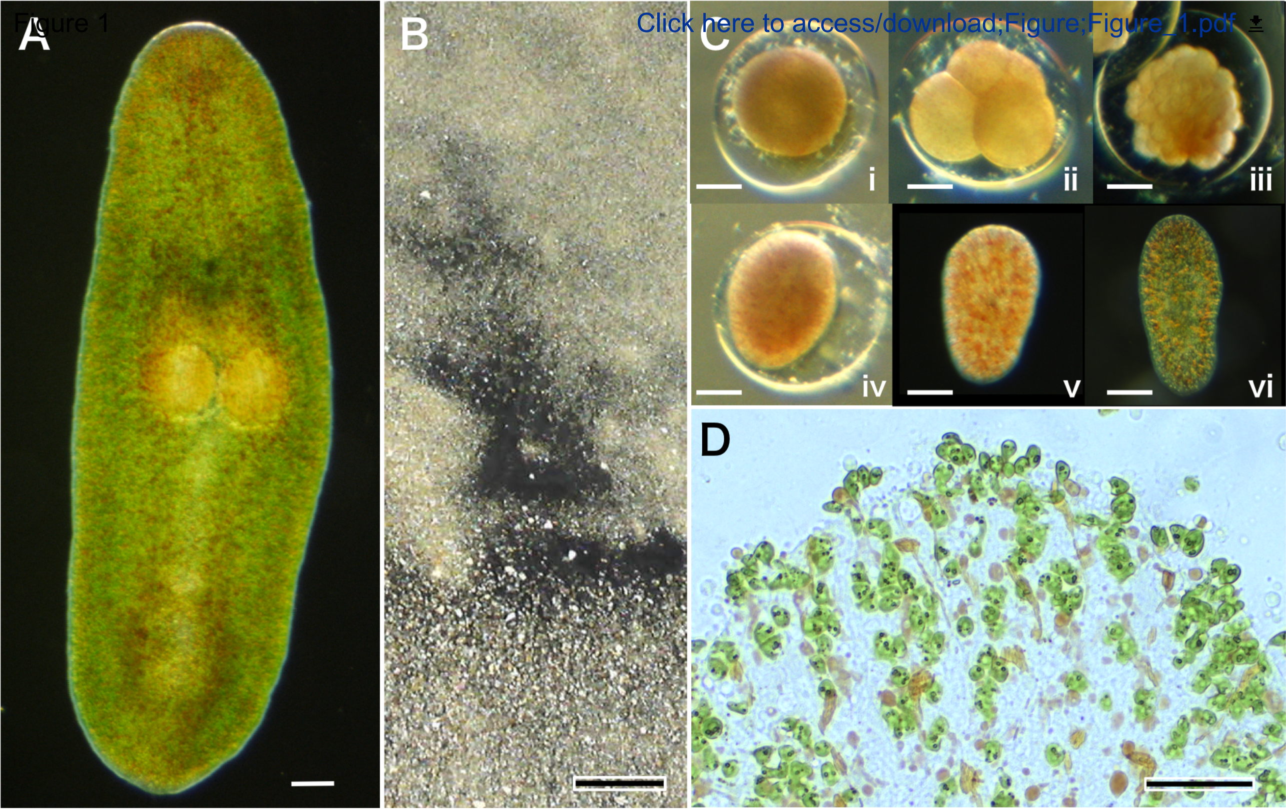

Figure 2

Click here to access/download;Figure;Figure 2.pdf

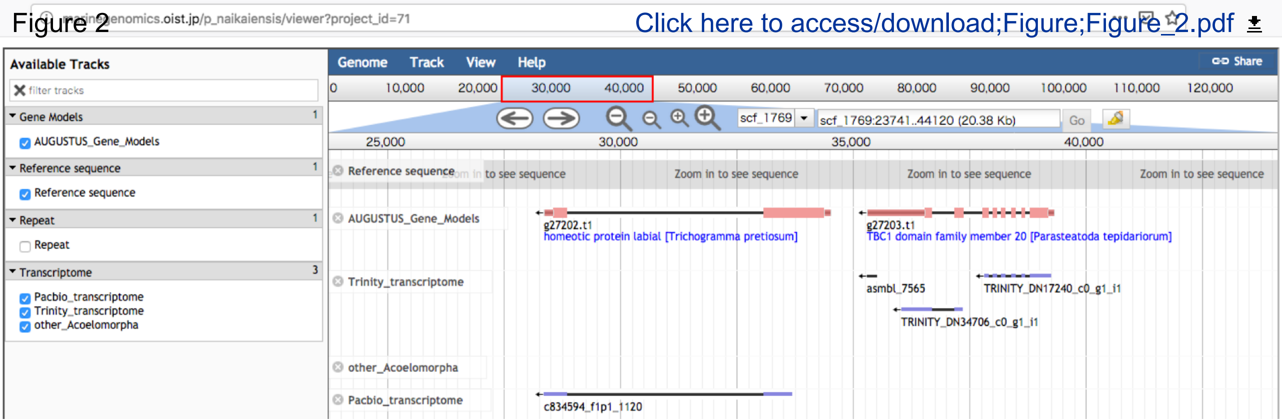

Supplementary Table 1

Click here to access/download
Supplementary Material
Table_S1.xlsx

Supplementary Figure 1

Click here to access/download
Supplementary Material
Figure_S1.pdf

Supplementary Table 2

Click here to access/download
Supplementary Material
Table_S2.xlsx

Supplementary Figure 2

Click here to access/download
Supplementary Material
Figure_S2.pdf

Supplementary Figure 3

Click here to access/download
Supplementary Material
Figure_S3.pdf

Supplementary Table 3

Click here to access/download
Supplementary Material
Table_S3.xlsx

Supplementary Figure 4

Click here to access/download
Supplementary Material
Figure_S4.pdf

Supplementary Figure 5

Click here to access/download
Supplementary Material
Figure_S5.pdf

Personal Cover Click here to access/download;Personal Cover;Response_Letter.190110.docx

GIGA-D-18-00363 (Revised form)

A draft genome assembly of the acoel flatworm *Praesagittifera naikaiensis*
Asuka Arimoto; Tomoe Hikosaka-Katayama; Akira Hikosaka; Kunifumi Tagawa; Toyoshige Inoue; Tatsuya Ueki; Masa-aki Yoshida; Miyuki Kanda; Eiichi Shoguchi; Kanako Hisata; Noriyuki Satoh

January 10, 2019
Dr. Hongling Zhou, Editor; *GigaScience*

Dear Dr. Zhou,

Thank you for your comments and those of two reviewers' regarding our manuscript "**A draft genome assembly of the acoel flatworm *Praesagittifera naikaiensis***" (GIGA-D-18-00363).

We are happy to receive positive and constructive comments from you and the reviewers. Accordingly, we have carefully revised the manuscript.

Perhaps due to the brevity of the description in the original version, we may have caused the reviewer #1 to misunderstand some points. Specifically, this is the first report of the nuclear genome assembly of acoels, although there are reports of acoel mitochondrial genomes. In the revised manuscript, we have clearly mentioned that we carried out a preliminary mitochondrial genome assembly to delete them from the nuclear genome assembly. We also mentioned that horizontal transfer of symbiotic microalgal genes into the acoel genome was not found in this study. You suggested a molecular phylogeny of acoels by adding our data. However, due to insufficiency of mitochondrial data of acoels to date, the resulting tree did not enjoy high bootstrap value (the tree is attached to the last page of this letter).

We submitted this manuscript as a "DATA NOTE", not a "RESEARCH" article. Our original version did not adequately explain the genome assembly, and, therefore, we here revised it according to the reviewers' comments. We also added the browser information in the text. Our hope is to publish the data (without a detailed analysis of the data) to facilitate studies of the genome of this interesting animal group. We appreciate your kind consideration of these research circumstances.

Hereafter we present a point-by-point response to reviewer comments.

Sincerely yours,

Asuka Arimoto, PhD

Marine Genomics Unit
Okinawa Institute of Science and Technology Graduate University (OIST)
1919-1, Tancha, Onna-Son, Kunigami, Okinawa 904-0495, Japan
Tel: +81-98-966-8653, FAX: +81-98-966-8622
asuka.arimoto@oist.jp

Reviewer reports:

Reviewer #1: The manuscript "A draft genome assembly of the acoel flatworm Praesagittifera naikaiensis" presents the 654 Mbp assembly for this flatworm. The genome appears to be assembled well, with good depth and using both Illumina and Pacbio reads for assembly, as well as RNA-seq for annotation.

(1-1) BUSCO analyses supported completeness of 77% of the annotated genes. BUSCO can also be ran against the genome assembly. This may be why your CEGMA numbers were substantially higher. Also, as reported in a recent study (https://www.nature.com/articles/s41588-018-0262-1/) there are some 7 "core" CEGMA genes that are consistently missing across all trematodes, suggesting that the BUSCO completeness may be higher than estimated, since there are likely some "core" functions that are legitimately absent from Praesagittifera naikaiensis. It may also be provided some "core" functions from its symbiosis with micro algae.

We appreciate the reviewer's comments. First, our BUSCO data were obtained by running BUSCO against the genome assembly. BUSCO analysis is carried out using metazoan genes while CEGMA against eukaryote genes. At present, we cannot explain the reason why the BUSCO score is lower than that of CEGMA, although they are similar. To avoid confusion between BUSCO and CEGMA results, we only used BUSCO analysis in the revised manuscript.

As to the comment that some core genes are consistently missing across all trematodes, our research group is now conducting a genome decoding project of a parasitic mesozoan, in which we found many lost genes in basic metabolic pathways. However, we failed to find such gene loss in this acoel genome.

Sorry but we cannot understand well the meaning of your comment, "It may also be provided some "core" functions from its symbiosis with micro algae". Regarding this, we carefully examined a possible mixture of algal genes in the acoel genome assembly. First, to avoid contamination of algal DNA, we used, as mentioned in "Biological materials", embryonic cells which do not contain symbiotic algae. Therefore, basically our data came from the acoel itself. Second, as you might mentioned, there is a possibility of horizontal transfer of microalgal genes into the acoel genome. In order to check whether the assembled genome contains sequences of photosynthetic organisms, we carried out blastx analysis of the assembled genome against NCBI NR database to find sequences with similarity to those of photosynthetic organisms. However, no such data were obtained. This convinces us that our draft assembly does not contain algal genes, although we have no idea at present on a possibility that some acoel core-functions depend on symbiotic algae.

(1-2) For tables 3 and 4, you could exclude all the entries with zero count.

Accordingly, we excluded the entries with zero counts from Tables 3 and 4.

(1-3) The genomes of *S. roscoffensis* and the xenoturbellid *X. bocki* are available. For the sake of evaluation and comparison of this genome, it would be very good to have a table comparing the basic statistics of these species (and any other xenacoelomorph species available), such as total length, protein coding genes, completeness, N50, etc. This would help to place this genome in the context of other available genomes and would help readers better connect resources in the future.

Probably due to the brevity of the description in the original version, we suspect that the reviewer misunderstood the present status of research in this field. That is, the present study is the first acoel "nuclear" genome, but not the first "mitochondrial" genome. Yes, there are reports of mitochondrial genomes of several acoel species, including *S. roscoffensis*, and *X. bocki* as well, but no nuclear genomes. Therefore, we cannot provide a table for genome comparison as the reviewer suggested. However, again, this is partially because our previous description was inadequate. We have revised the manuscript to distinguish clearly between nuclear and mitochondrial genomes (pages 2, 3, 5, and 7).

Reviewer #2: The authors collected genomic and transcriptomic data for the acoelomate worm Praesagittifera naikaiensis. The species belongs to an important group of organisms that are key to understanding the origin of bilateral body plan, the ability of whole-body regeneration, and symbiosis with photosynthetic microalgae. Genomic resources for this an organism will help these key areas of research.

The authors used Pacific Biosciences long reads and Illumina paired end short reads for both genomic and transcriptomic data sets. They used a hybrid approach for de novo assembly and Iso-seq for validation of the transcripts predicted with the RNAseq data. I have some minor concerns and suggestions regarding the assembly approach and presentation of the paper:

(2-1) The authors collected high coverage (73X) PacBio reads for genome assembly. At this coverage, a PacBio only assembler is likely to produce a more contiguous and accurate assembly (e.g. see https://academic.oup.com/nar/article/44/19/e147/2468393). Given that a heterozygous sample was sequenced, Falcon could be used as the PacBio only assembler. I was also wondering if the authors tried the hybrid assembler DBG2OLC (and Platanus as the Illumina assembler as described in https://academic.oup.com/nar/article/44/19/e147/2468393) which often works better than Masurca?

We appreciate the reviewer's comments on the methodology of genome assembly. Our research group has so far sequenced genomes of more than 10 animal taxa. The assembly is affected by the choice of Illumina and/or PacBio platform, or their combination; therefore, we examined various methods including those the reviewer suggested. For example, we tried the FALCON assembler using subreads with more than 2 kb of PacBio, but the total assembled length resulted in only 2.6 Mb. We have obtained 73X PacBio data, but those more than 5 kb were only 20X. Another cause might come from sampling of embryos from different batches (it is impossible to obtain enough samples from a single individual). We also attempted to use a hybrid assembler DBG2OLC with Platanus to obtain a better contig assembly. The most suitable parameter usage gave rise to a 630 Mb assembly with scaffold number 11 million and scaffold N50 = 50 bp. Namely, compared to MaSuRCA, all these scaffolds were very fragmented.

(2-2) The authors used Racon to polish the assembly with long reads. However, Quiver or Arrow is recommended over Racon for polishing PacBio assemblies. With 70X coverage, Arrow (and Quiver) can achieve higher consensus accuracy than Racon.

As mentioned above, probably due to mixed embryonic samples from different batches, our PacBio reads did not always provide data useful for further analysis, such as with Arrow. However, as mentioned above, we tried various polishing methods, and Racon combined with Pilon resulted in the best assembly; thus, we presented data resulting from this method.

(2-3) On line 195, "others" is mentioned as if it is a type of TE. It would be more appropriate to mention them as unclassified. On a related note, all repeats appear to consist of only TEs. Do these worms not have any simple or Low complexity repeats?

We appreciate this comment. Accordingly, we changed the description of “others” to more explicit language, including simple repeats. We also explained more clearly the rate and types of TEs. (Page 8, lines 205-215)

(2-4) The statements on the relationships between single copy and double copy genes and BUSCO and CEGMA were unclear (Line 201-204). The BUSCO and CEGMA both report the single copy and double copy genes based on their database and the percentages are based on the number of conserved genes they have searched from their database. It would be helpful to clarify these.

Good comment. Accordingly, we revised Table 1 (more simple form), in which “single and double copy genes” were deleted. In the revised form, we removed the CEGMA data in order to avoid confusion between BUSCO and CEGMA data. (Page 9, lines 231-232)

(2-5) One interesting analysis that the authors could do is to check the number of TEs that are located within the introns and the number of introns that are only TEs (intron length = TE length).

This was an interesting suggestion so that we checked it. We found that 32,110 TEs are present in intron regions; 29%, 18%, and 12% of them are correspond to “uncharacterized”, “LTR (Gypsy)” and DNA transposon (MULE), respectively. On the other hand, we failed to find introns that comprise only TEs. We have now included this result. (Page 8, lines 216-220).

(2-6) The authors mention that the adult worms carry symbiotic algae. I am curious to know whether the authors found any sequence reads that are derived from symbiotic algae. It would be nice to get this information. Similarly, does any of the contigs belong to symbiotic algae?

As the reviewer pointed out, the adult worms carry symbiotic algae. To avoid contamination from algal DNA, we used embryonic cells that do not contain symbiotic algae. Therefore, basically our data came from the acoel itself. However, as you mentioned, there is a possibility of horizontal transfer of microalgal genes into the acoel genome or contamination of algae

during sampling procedure. In order to check whether the assembled genome contains sequences of photosynthetic organisms, we carried out blastx analysis of the assembled genome against NCBI NR database to find sequences with similarity to those of photosynthetic organisms. However, no such data were obtained. This convinces us that our draft assembly does not contain algal genes.

(2-7) I could not access the genome browser at the marinegenomics website the authors have provided. Is the link correct?

We apologize for the inconvenience. We will open the genome browser if our manuscript is accepted. However, an account for reviewing is available now. Reviewers can login to the browser at http://marinegenomics.oist.jp/gallery/users/sign_in with account ID: acoel-pna, and password: acoel-genome. We added in the revised form more clearly the genome browser information (Figure 2).

(2-8) On Line 162, the sentence that starts with "parallel" looks incomplete and needs to be revised.

Sorry. We changed “Parallel” to “In addition,” (line 165).

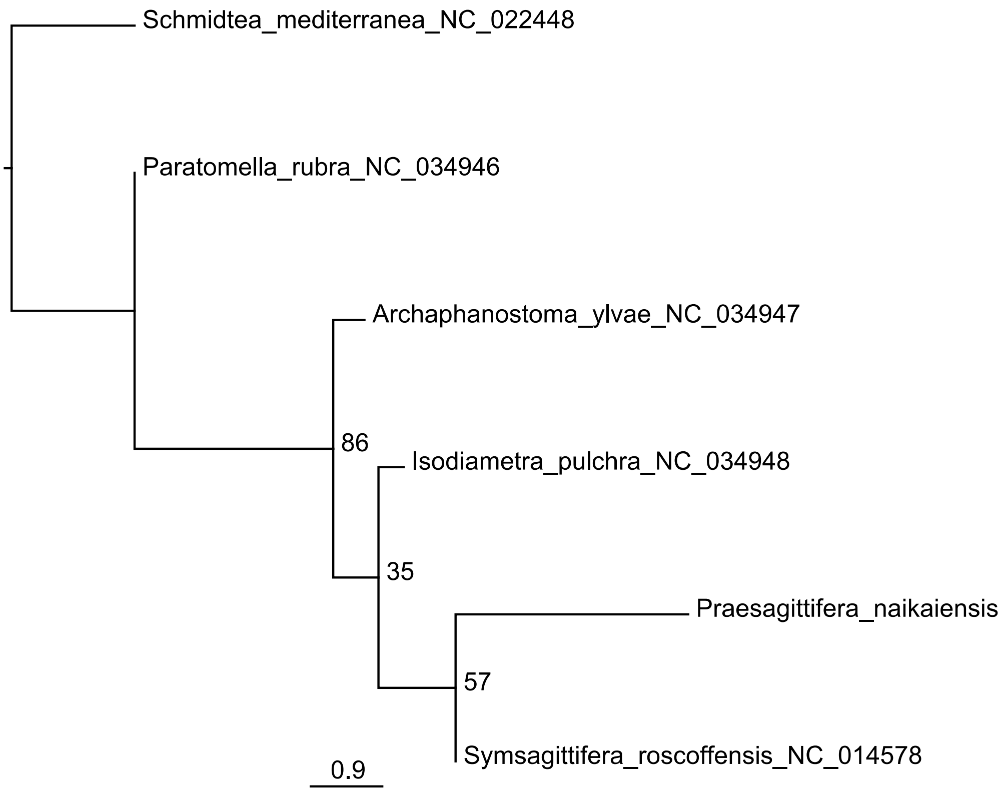

Appendix 1. Phylogenetic tree of acoels. The tree was constructed with the maximum-likelihood method using 10 mitochondrial protein-coding gene sequences; a planarian (*Schmidtea*) as outgroup. Numbers at nodes represent bootstrap values. The scale bar shows the number of substitutions per site.
