# Supplementary material for: A draft nuclear-genome assembly of the acoel flatworm Praesagittifera naikaiensis
Source: Gigascience. 2019 Apr 6;8(4):giz023. doi: 10.1093/gigascience/giz023 (PMC6451197; doi:10.1093/gigascience/giz023)
Supplement: Supplemental Files [file giz023_supplemental_files.zip › Figure_S1.pdf]

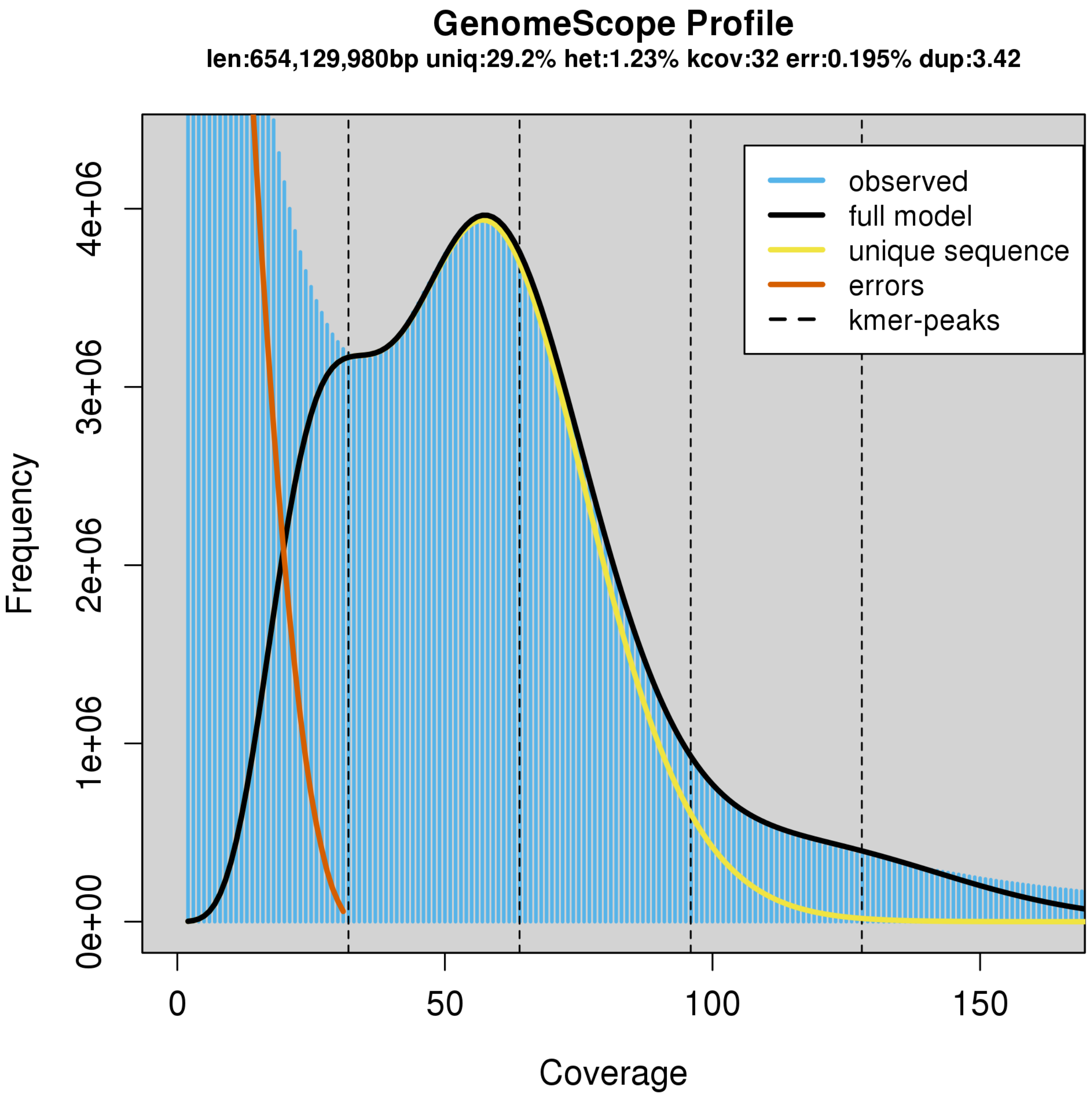

Supplementary Figure 1: K-mer analysis and genome size estimation of *Praesagittifera naikaiensis* genomic DNA reads. X-axis shows coverage and Y-axis shows frequency.
