# Supplementary material for: A draft nuclear-genome assembly of the acoel flatworm Praesagittifera naikaiensis
Source: Gigascience. 2019 Apr 6;8(4):giz023. doi: 10.1093/gigascience/giz023 (PMC6451197; doi:10.1093/gigascience/giz023)
Supplement: Supplemental Files [file giz023_supplemental_files.zip › Figure_S2.pdf]

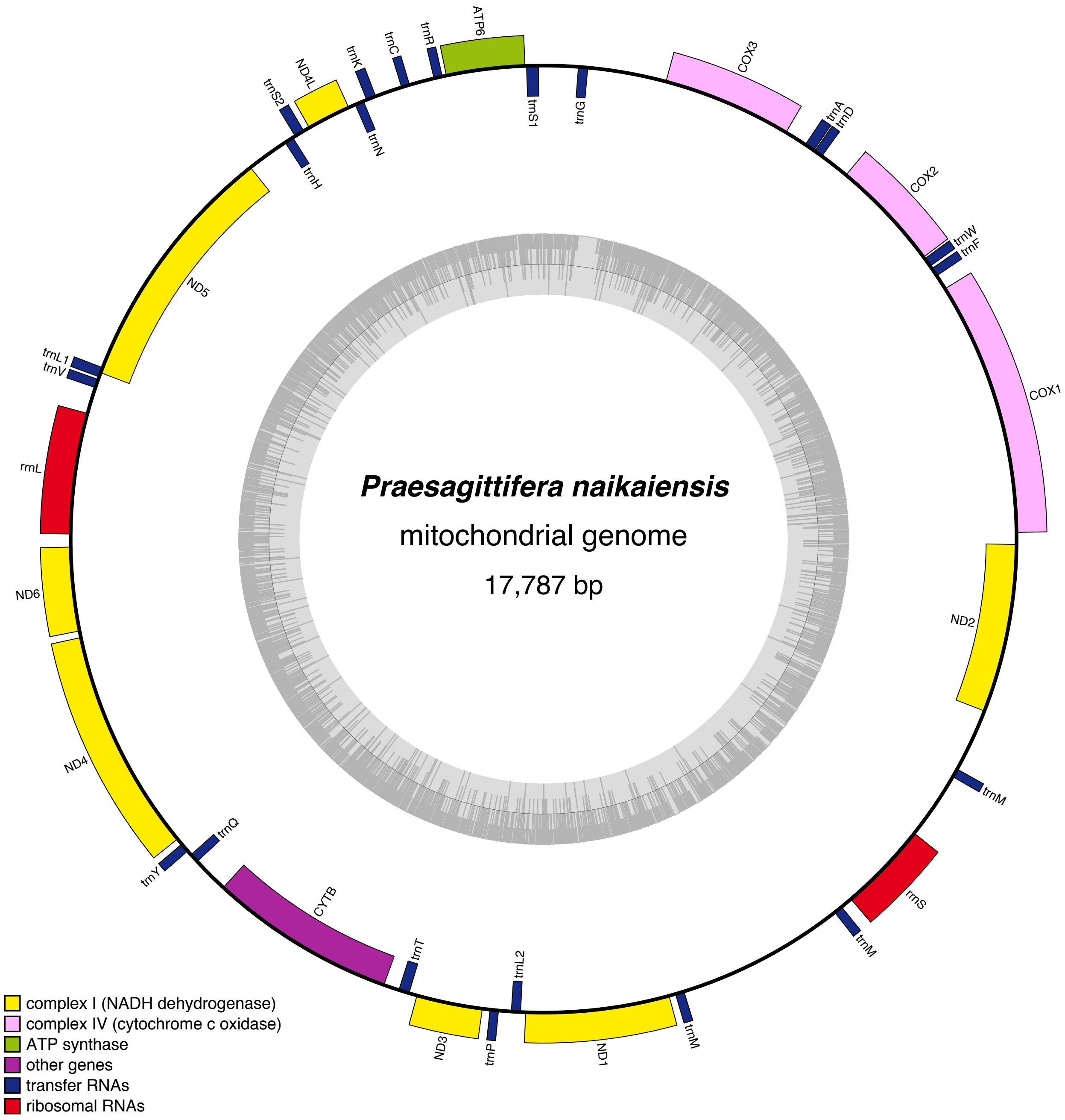


**Supplementary Figure 2: A preliminary circular assembly of the mitochondrial genome of *Praesagittifera naikaiensis*.** The mitochondrial genome contains 12 protein-coding genes, two rRNAs and 22 predicted tRNAs.
