# Supplementary material for: A draft nuclear-genome assembly of the acoel flatworm Praesagittifera naikaiensis
Source: Gigascience. 2019 Apr 6;8(4):giz023. doi: 10.1093/gigascience/giz023 (PMC6451197; doi:10.1093/gigascience/giz023)
Supplement: Supplemental Files [file giz023_supplemental_files.zip › Figure_S3.pdf]

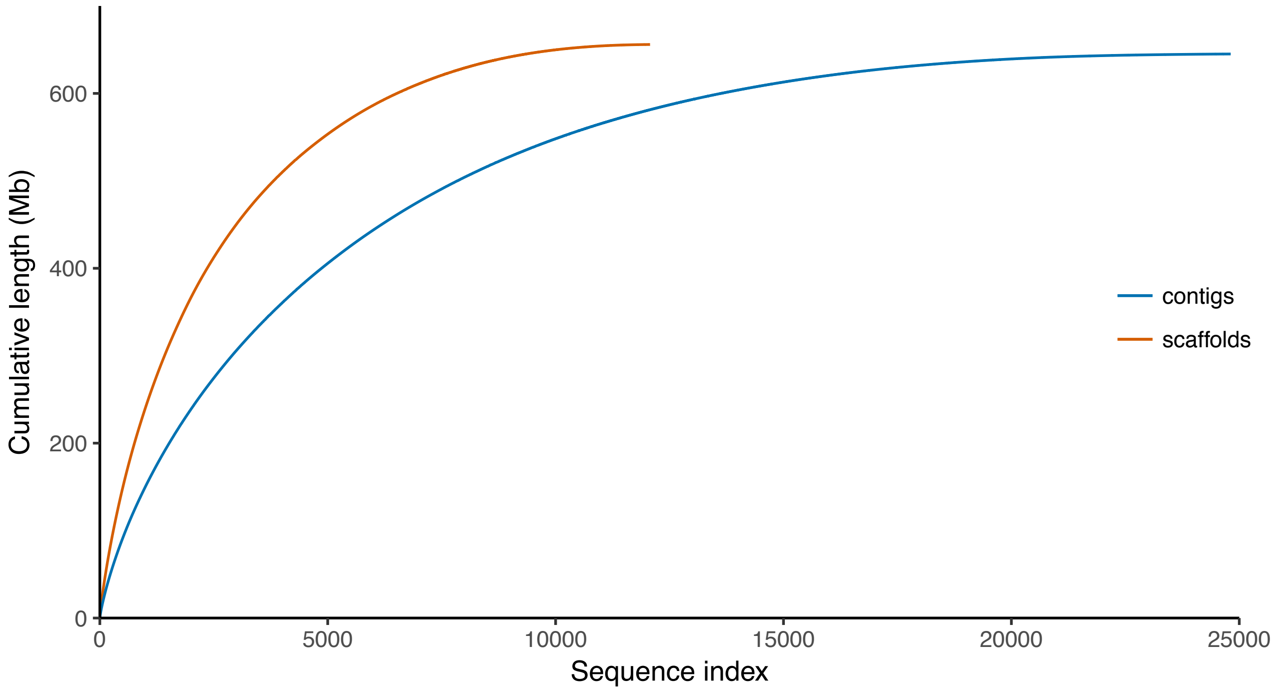

Supplementary Figure 3: Accumulation of assembled sequences (contigs, blue and scaffolds, red) reaching over 600 Mb. X-axis shows sequence index and Y-axis shows cumulative length (Mb).
