# Supplementary material for: A draft nuclear-genome assembly of the acoel flatworm Praesagittifera naikaiensis
Source: Gigascience. 2019 Apr 6;8(4):giz023. doi: 10.1093/gigascience/giz023 (PMC6451197; doi:10.1093/gigascience/giz023)
Supplement: Supplemental Files [file giz023_supplemental_files.zip › Figure_S5.pdf]

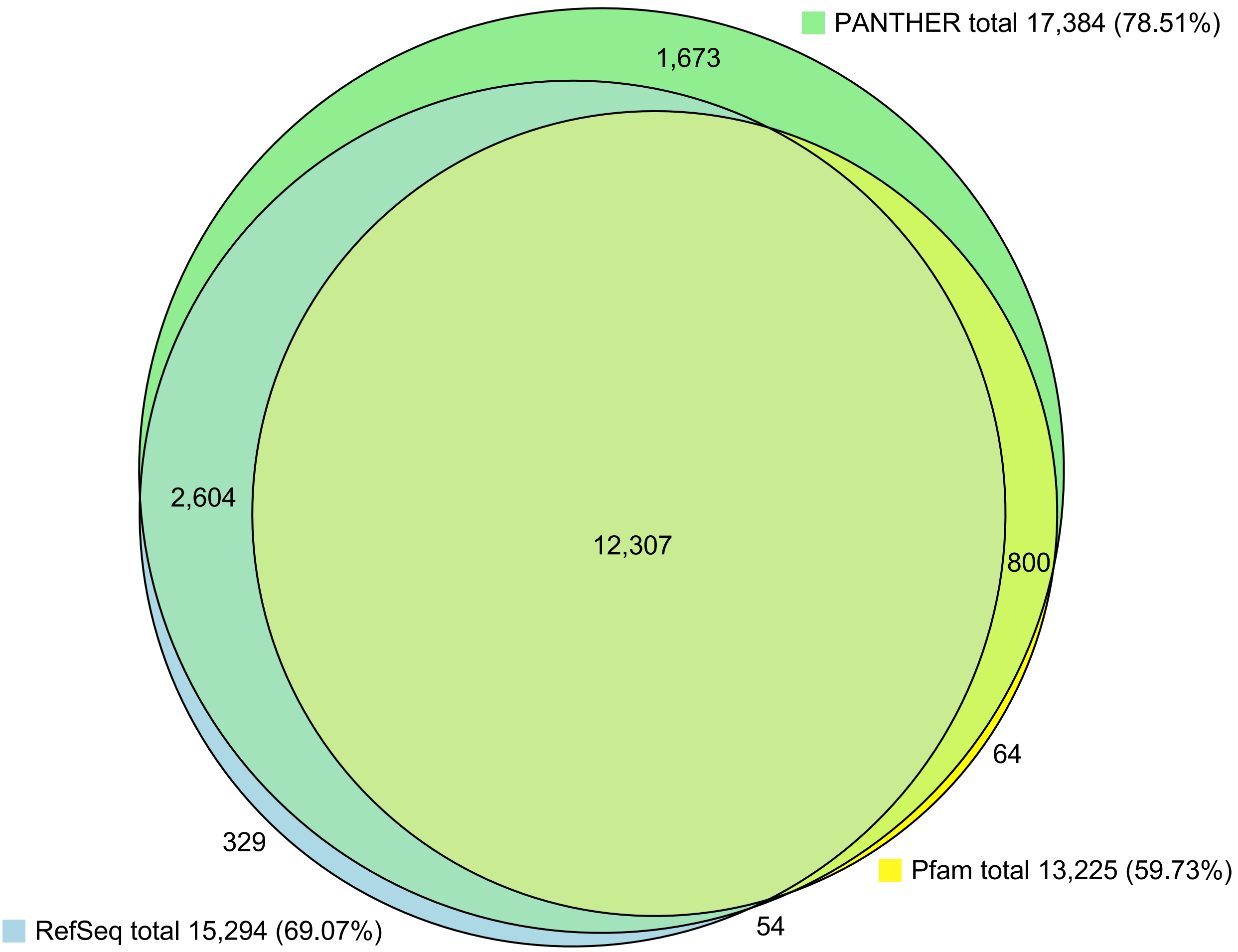

Supplementary Figure 5: *Praesagittifera naikaiensis* gene annotation. A total of 22,143 genes were annotated by different databases.
