# Supplementary material for: A draft nuclear-genome assembly of the acoel flatworm Praesagittifera naikaiensis
Source: Gigascience. 2019 Apr 6;8(4):giz023. doi: 10.1093/gigascience/giz023 (PMC6451197; doi:10.1093/gigascience/giz023)
Supplement: Response_to_Reviewer_Comments_Original_Submission.pdf [file giz023_response_to_reviewer_comments_original_submission.pdf]

Author's Response To Reviewer Comments

Close

Reviewer reports:

Reviewer #1: The manuscript "A draft genome assembly of the acoel flatworm Praesagittifera naikaiensis" presents the 654 Mbp assembly for this flatworm. The genome appears to be assembled well, with good depth and using both Illumina and Pacbio reads for assembly, as well as RNA-seq for annotation.

(1-1) BUSCO analyses supported completeness of 77% of the annotated genes. BUSCO can also be ran against the genome assembly. This may be why your CEGMA numbers were substantially higher. Also, as reported in a recent study (https://www.nature.com/articles/s41588-018-0262-1/) there are some 7 "core" CEGMA genes that are consistently missing across all trematodes, suggesting that the BUSCO completeness may be higher than estimated, since there are likely some "core" functions that are legitimately absent from Praesagittifera naikaiensis. It may also be provided some "core" functions from its symbiosis with micro algae.

We appreciate the reviewer's comments. First, our BUSCO data were obtained by running BUSCO against the genome assembly. BUSCO analysis is carried out using metazoan genes while CEGMA against eukaryote genes. At present, we cannot explain the reason why the BUSCO score is lower than that of CEGMA, although they are similar. To avoid confusion between BUSCO and CEGMA results, we only used BUSCO analysis in the revised manuscript.
As to the comment that some core genes are consistently missing across all trematodes, our research group is now conducting a genome decoding project of a parasitic mesozoan, in which we found many lost genes in basic metabolic pathways. However, we failed to find such gene loss in this acoel genome.
Sorry but we cannot understand well the meaning of your comment, "It may also be provided some "core" functions from its symbiosis with micro algae". Regarding this, we carefully examined a possible mixture of algal genes in the acoel genome assembly. First, to avoid contamination of algal DNA, we used, as mentioned in "Biological materials", embryonic cells which do not contain symbiotic algae. Therefore, basically our data came from the acoel itself. Second, as you might mentioned, there is a possibility of horizontal transfer of microalgal genes into the acoel genome. In order to check whether the assembled genome contains sequences of photosynthetic organisms, we carried out blastx analysis of the assembled genome against NCBI NR database to find sequences with similarity to those of photosynthetic organisms. However, no such data were obtained. This convinces us that our draft assembly does not contain algal genes, although we have no idea at present on a possibility that some acoel core-functions depend on symbiotic algae.

(1-2) For tables 3 and 4, you could exclude all the entries with zero count.

Accordingly, we excluded the entries with zero counts from Tables 3 and 4.

(1-3) The genomes of S. roscoffensis and the xenoturbellid X. bocki are available. For the sake of evaluation and comparison of this genome, it would be very good to have a table comparing the basic statistics of these species (and any other xenacoelomorph species available), such as total length, protein coding genes, completeness, N50, etc. This would help to place this genome in the context of other available genomes and would help readers better connect resources in the future.

Probably due to the brevity of the description in the original version, we suspect that the reviewer misunderstood the present status of research in this field. That is, the present study is the first acoel "nuclear" genome, but not the first "mitochondrial" genome. Yes, there are reports of mitochondrial

genomes of several acoel species, including S. roscoffensis, and X. bocki as well, but no nuclear genomes. Therefore, we cannot provide a table for genome comparison as the reviewer suggested. However, again, this is partially because our previous description was inadequate. We have revised the manuscript to distinguish clearly between nuclear and mitochondrial genomes (pages 2, 3, 5, and 7).

Reviewer #2: The authors collected genomic and transcriptomic data for the acoelomate worm Praesagittifera naikaiensis. The species belongs to an important group of organisms that are key to understanding the origin of bilateral body plan, the ability of whole-body regeneration, and symbiosis with photosynthetic microalgae. Genomic resources for this an organism will help these key areas of research.

The authors used Pacific Biosciences long reads and Illumina paired end short reads for both genomic and transcriptomic data sets. They used a hybrid approach for de novo assembly and Iso-seq for validation of the transcripts predicted with the RNAseq data. I have some minor concerns and suggestions regarding the assembly approach and presentation of the paper:

(2-1) The authors collected high coverage (73X) PacBio reads for genome assembly. At this coverage, a PacBio only assembler is likely to produce a more contiguous and accurate assembly (e.g. see https://academic.oup.com/nar/article/44/19/e147/2468393). Given that a heterozygous sample was sequenced, Falcon could be used as the PacBio only assembler. I was also wondering if the authors tried the hybrid assembler DBG2OLC (and Platanus as the Illumina assembler as described in https://academic.oup.com/nar/article/44/19/e147/2468393) which often works better than Masurca?

We appreciate the reviewer’s comments on the methodology of genome assembly. Our research group has so far sequenced genomes of more than 10 animal taxa. The assembly is affected by the choice of Illumina and/or PacBio platform, or their combination; therefore, we examined various methods including those the reviewer suggested. For example, we tried the FALCON assembler using subreads with more than 2 kb of PacBio, but the total assembled length resulted in only 2.6 Mb. We have obtained 73X PacBio data, but those more than 5 kb were only 20X. Another cause might come from sampling of embryos from different batches (it is impossible to obtain enough samples from a single individual). We also attempted to use a hybrid assembler DBG2OLC with Platanus to obtain a better contig assembly. The most suitable parameter usage gave rise to a 630 Mb assembly with scaffold number 11 million and scaffold N50 = 50 bp. Namely, compared to MaSuRCA, all these scaffolds were very fragmented.

(2-2) The authors used Racon to polish the assembly with long reads. However, Quiver or Arrow is recommended over Racon for polishing PacBio assemblies. With 70X coverage, Arrow (and Quiver) can achieve higher consensus accuracy than Racon.

As mentioned above, probably due to mixed embryonic samples from different batches, our PacBio reads did not always provide data useful for further analysis, such as with Arrow. However, as mentioned above, we tried various polishing methods, and Racon combined with Pilon resulted in the best assembly; thus, we presented data resulting from this method.

(2-3) On line 195, "others" is mentioned as if it is a type of TE. It would be more appropriate to mention them as unclassified. On a related note, all repeats appear to consist of only TEs. Do these worms not have any simple or Low complexity repeats?

We appreciate this comment. Accordingly, we changed the description of “others” to more explicit language, including simple repeats. We also explained more clearly the rate and types of TEs. (Page 8, lines 205-215)

(2-4) The statements on the relationships between single copy and double copy genes and BUSCO and CEGMA were unclear (Line 201-204). The BUSCO and CEGMA both report the single copy and double copy genes based on their database and the percentages are based on the number of conserved genes they

have searched from their database. It would be helpful to clarify these.

Good comment. Accordingly, we revised Table 1 (more simple form), in which “single and double copy genes” were deleted. In the revised form, we removed the CEGMA data in order to avoid confusion between BUSCO and CEGMA data. (Page 9, lines 231-232)

(2-5) One interesting analysis that the authors could do is to check the number of TEs that are located within the introns and the number of introns that are only TEs (intron length = TE length).

This was an interesting suggestion so that we checked it. We found that 32,110 TEs are present in intron regions; 29%, 18%, and 12% of them are correspond to “uncharacterized”, “LTR (Gypsy)” and DNA transposon (MULE), respectively. On the other hand, we failed to find introns that comprise only TEs. We have now included this result. (Page 8, lines 216-220).

(2-6) The authors mention that the adult worms carry symbiotic algae. I am curious to know whether the authors found any sequence reads that are derived from symbiotic algae. It would be nice to get this information. Similarly, does any of the contigs belong to symbiotic algae?

As the reviewer pointed out, the adult worms carry symbiotic algae. To avoid contamination from algal DNA, we used embryonic cells that do not contain symbiotic algae. Therefore, basically our data came from the acoel itself. However, as you mentioned, there is a possibility of horizontal transfer of microalgal genes into the acoel genome or contamination of algae during sampling procedure. In order to check whether the assembled genome contains sequences of photosynthetic organisms, we carried out blastx analysis of the assembled genome against NCBI NR database to find sequences with similarity to those of photosynthetic organisms. However, no such data were obtained. This convinces us that our draft assembly does not contain algal genes.

(2-7) I could not access the genome browser at the marinegenomics website the authors have provided. Is the link correct?

We apologize for the inconvenience. We will open the genome browser if our manuscript is accepted. However, an account for reviewing is available now. Reviewers can login to the browser at http://marinegenomics.oist.jp/gallery/users/sign_in with account ID: acoel-pna, and password: acoel-genome. We added in the revised form more clearly the genome browser information (Figure 2).

(2-8) On Line 162, the sentence that starts with "parallel" looks incomplete and needs to be revised.

Sorry. We changed “Parallel” to “In addition,” (line 165).

Close
