# Supplementary material for: A draft nuclear-genome assembly of the acoel flatworm Praesagittifera naikaiensis
Source: Gigascience. 2019 Apr 6;8(4):giz023. doi: 10.1093/gigascience/giz023 (PMC6451197; doi:10.1093/gigascience/giz023)
Supplement: Reviewer_1_Report_Original_Submission -- Bruce A Rosa — 11/10/2018 Reviewed [file giz023_reviewer_1_report_original_submission.pdf]

Reviewer Report

Title: A draft genome assembly of the acoel flatworm Praesagittifera naikaiensis

Version: Original Submission **Date:** 11/10/2018

Reviewer name: Bruce A Rosa

Reviewer Comments to Author:

The manuscript "A draft genome assembly of the acoel flatworm Praesagittifera naikaiensis" presents the 654 Mbp assembly for this flatworm. The genome appears to be assembled well, with good depth and using both Illumina and Pacbio reads for assembly, as well as RNA-seq for annotation.
- BUSCO analyses supported completeness of 77% of the annotated genes. BUSCO can also be ran against the genome assembly. This may be why your CEGMA numbers were substantially higher. Also, as reported in a recent study (https://www.nature.com/articles/s41588-018-0262-1/) there are some 7 "core" CEGMA genes that are consistently missing across all trematodes, suggesting that the BUSCO completeness may be higher than estimated, since there are likely some "core" functions that are legitimately absent from Praesagittifera naikaiensis. It may also be provided some "core" functions from its symbiosis with micro algae.
- For tables 3 and 4, you could exclude all the entries with zero count.
- The genomes of S. roscoffensis and the xenoturbellid X. bocki are available. For the sake of evaluation and comparison of this genome, it would be very good to have a table comparing the basic statistics of these species (and any other Xenacoelomorpha species available), such as total length, protein coding genes, completeness, N50, etc. This would help to place this genome in the context of other available genomes, and help readers to better connect the resources in the future.

Level of Interest

Please indicate how interesting you found the manuscript: Choose an item.

Quality of Written English

Please indicate the quality of language in the manuscript: Choose an item.

Declaration of Competing Interests

Please complete a declaration of competing interests, considering the following questions:

- Have you in the past five years received reimbursements, fees, funding, or salary from an organisation that may in any way gain or lose financially from the publication of this manuscript, either now or in the future?
- Do you hold any stocks or shares in an organisation that may in any way gain or lose financially from the publication of this manuscript, either now or in the future?

- Do you hold or are you currently applying for any patents relating to the content of the manuscript?
- Have you received reimbursements, fees, funding, or salary from an organization that holds or has applied for patents relating to the content of the manuscript?
- Do you have any other financial competing interests?
- Do you have any non-financial competing interests in relation to this paper?

If you can answer no to all of the above, write 'I declare that I have no competing interests' below. If your reply is yes to any, please give details below.

I declare that I have no competing interests

I agree to the open peer review policy of the journal. I understand that my name will be included on my report to the authors and, if the manuscript is accepted for publication, my named report including any attachments I upload will be posted on the website along with the authors' responses. I agree for my report to be made available under an Open Access Creative Commons CC-BY license (http://creativecommons.org/licenses/by/4.0/). I understand that any comments which I do not wish to be included in my named report can be included as confidential comments to the editors, which will not be published.

I agree to the open peer review policy of the journal

To further support our reviewers, we have joined with Publons, where you can gain additional credit to further highlight your hard work (see: https://publons.com/journal/530/gigascience). On publication of this paper, your review will be automatically added to Publons, you can then choose whether or not to claim your Publons credit. I understand this statement.

Yes Choose an item.
