# Supplementary material for: A draft nuclear-genome assembly of the acoel flatworm Praesagittifera naikaiensis
Source: Gigascience. 2019 Apr 6;8(4):giz023. doi: 10.1093/gigascience/giz023 (PMC6451197; doi:10.1093/gigascience/giz023)
Supplement: Reviewer_2_Report_Original_Submission -- Mahul Chakraborty — 11/22/2018 Reviewed [file giz023_reviewer_2_report_original_submission.pdf]

Reviewer Report

Title: A draft genome assembly of the acoel flatworm Praesagittifera naikaiensis

Version: Original Submission **Date:** 11/22/2018

Reviewer name: Mahul Chakraborty

Reviewer Comments to Author:

The authors collected genomic and transcriptomic data for the acoelomate worm Praesagittifera naikaiensis. The species belongs to an important group of organisms that are key to understanding the origin of bilateral body plan, the ability of whole body regeneration, and symbiosis with photosynthetic microalgae. Genomic resources for this an organism will help these key areas of research.
The authors used Pacific Biosciences long reads and Illumina paired end short reads for both genomic and transcriptomic data sets. They used a hybrid approach for de novo assembly and Iso-seq for validation of the transcripts predicted with the RNAseq data. I have some minor concerns and suggestions regarding the assembly approach and presentation of the paper:
The authors collected high coverage (73X) PacBio reads for genome assembly. At this coverage, a PacBio only assembler is likely to produce a more contiguous and accurate assembly (e.g. see https://academic.oup.com/nar/article/44/19/e147/2468393). Given that a heterozygous sample was sequenced, Falcon could be used as the PacBio only assembler. I was also wondering if the authors tried the hybrid assembler DBG2OLC (and Platanus as the Illumina assembler as described in https://academic.oup.com/nar/article/44/19/e147/2468393) which often works better than Masurca?
The authors used Racon to polish the assembly with long reads. However, Quiver or Arrow is recommended over Racon for polishing PacBio assemblies. With 70X coverage, Arrow (and Quiver) can achieve higher consensus accuracy than Racon.
On line 195, "others" is mentioned as if it is a type of TE. It would be more appropriate to mention them as unclassified. On a related note, all repeats appear to consist of only TEs. Do these worms not have any simple or Low complexity repeats ?
The statements on the relationships between single copy and double copy genes and BUSCO and CEGMA were unclear (Line 201-204). The BUSCO and CEGMA both report the single copy and double copy genes based on their database and the percentages are based on the the number of conserved genes they have searched from their database. It would be helpful to clarify these.
One interesting analysis that the authors could do is to check the number of TEs that are located within the introns and the number of introns that are only TEs (intron length = TE length).
The authors mention that the adult worms carry symbiotic algae. I am curious to know whether the authors found any sequence reads that are derived from symbiotic algae. It would be nice to get this information. Similarly, does any of the contigs belong to symbiotic algae?
I could not access the genome browser at the marinegenomics website the authors have provided. Is the link correct?
On Line 162, the sentence that starts with "parallel" looks incomplete and needs to be revised.

Level of Interest

Please indicate how interesting you found the manuscript: Choose an item.

Quality of Written English

Please indicate the quality of language in the manuscript: Choose an item.

Declaration of Competing Interests

Please complete a declaration of competing interests, considering the following questions:

- Have you in the past five years received reimbursements, fees, funding, or salary from an organisation that may in any way gain or lose financially from the publication of this manuscript, either now or in the future?
- Do you hold any stocks or shares in an organisation that may in any way gain or lose financially from the publication of this manuscript, either now or in the future?
- Do you hold or are you currently applying for any patents relating to the content of the manuscript?
- Have you received reimbursements, fees, funding, or salary from an organization that holds or has applied for patents relating to the content of the manuscript?
- Do you have any other financial competing interests?
- Do you have any non-financial competing interests in relation to this paper?

If you can answer no to all of the above, write 'I declare that I have no competing interests' below. If your reply is yes to any, please give details below.

I declare that I have no competing interests

I agree to the open peer review policy of the journal. I understand that my name will be included on my report to the authors and, if the manuscript is accepted for publication, my named report including any attachments I upload will be posted on the website along with the authors' responses. I agree for my report to be made available under an Open Access Creative Commons CC-BY license (http://creativecommons.org/licenses/by/4.0/). I understand that any comments which I do not wish to be included in my named report can be included as confidential comments to the editors, which will not be published.

I agree to the open peer review policy of the journal

To further support our reviewers, we have joined with Publons, where you can gain additional credit to further highlight your hard work (see: https://publons.com/journal/530/gigascience). On publication of this paper, your review will be automatically added to Publons, you can then choose whether or not to claim your Publons credit. I understand this statement.

Yes Choose an item.
